# Long-Term Protective Effect of Human Dystrophin Expressing Chimeric (DEC) Cell Therapy on Amelioration of Function of Cardiac, Respiratory and Skeletal Muscles in Duchenne Muscular Dystrophy

**DOI:** 10.1007/s12015-022-10384-2

**Published:** 2022-05-19

**Authors:** Maria Siemionow, Paulina Langa, Sonia Brodowska, Katarzyna Kozlowska, Kristina Zalants, Katarzyna Budzynska, Ahlke Heydemann

**Affiliations:** 1 Department of Orthopaedics, University of Illinois at Chicago, Chicago, IL, USA; 2 Department of Surgery, Poznan University of Medical Science, Poznan, Poland; 3 Department of Physiology and Biophysics, University of Illinois at Chicago, Chicago, IL USA

**Keywords:** Duchenne Muscular Dystrophy, Stem cells, Systemic-intraosseous administration, DEC therapy, Dystrophin, Cell fusion, Chimeric cells, DMD, Transplant, *mdx/scid* mice, ATMP

## Abstract

Duchenne Muscular Dystrophy (DMD) is a lethal disease caused by mutations in dystrophin encoding gene, causing progressive degeneration of cardiac, respiratory, and skeletal muscles leading to premature death due to cardiac and respiratory failure. Currently, there is no cure for DMD. Therefore, novel therapeutic approaches are needed for DMD patients.

We have previously reported functional improvements which correlated with increased dystrophin expression following administration of dystrophin expressing chimeric (DEC) cells of myoblast origin to the *mdx* mouse models of DMD.

In the current study, we confirmed dose-dependent protective effect of human DEC therapy created from myoblasts of normal and DMD-affected donors, on restoration of dystrophin expression and amelioration of cardiac, respiratory, and skeletal muscle function at 180 days after systemic-intraosseous DEC administration to *mdx/scid* mouse model of DMD. Functional improvements included maintenance of ejection fraction and fractional shortening levels on echocardiography, reduced enhanced pause and expiration time on plethysmography and improved grip strength and maximum stretch induced contraction of skeletal muscles. Improved function was associated with amelioration of *mdx* muscle pathology revealed by reduced muscle fibrosis, reduced inflammation and improved muscle morphology confirmed by reduced number of centrally nucleated fibers and normalization of muscle fiber diameters. Our findings confirm the long-term systemic effect of DEC therapy in the most severely affected by DMD organs including heart, diaphragm, and long skeletal muscles.

These encouraging preclinical data introduces human DEC as a novel therapeutic modality of Advanced Therapy Medicinal Product (ATMP) with the potential to improve or halt the progression of DMD and enhance quality of life of DMD patients.

**Graphical Abstract:**

Human DEC as a novel therapeutic modality with the potential to improve or halt progression of the DMD disease and enhance quality of life of DMD patients. Graphical abstract represents manufacturing process of the human DEC therapy for the future clinical applications. 1. We report the long-term efficacy of human DEC therapy resulting in increased dystrophin expression and reduced *mdx* muscle pathology after systemic-intraosseous administration of human Dystrophin Expressing Chimeric (DEC) Cells to the *mdx/scid* mouse model of DMD. 2. Systemic administration of human DEC therapy resulted in amelioration of cardiac, respiratory and skeletal muscle function as confirmed by echocardiography, plethysmography and standard muscle strength tests respectively. 3. We introduce human DEC as a novel Advanced Therapy Medicinal Product (ATMP) for future clinical application in DMD patients.

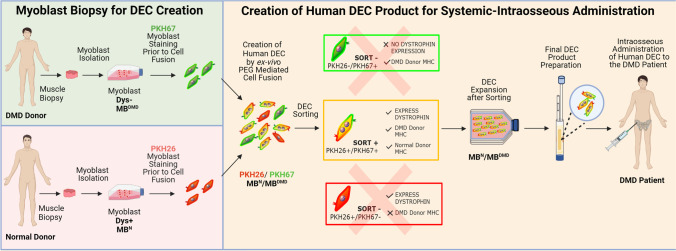

**Supplementary Information:**

The online version contains supplementary material available at 10.1007/s12015-022-10384-2.

## Background

Duchenne Muscular Dystrophy is an X-linked recessive neuromuscular disorder caused by mutations in the dystrophin gene with the incidence of approximately 1 in 3,500–5,000 newborn boys [1].

Due to the lack of dystrophin, a large structural protein that stabilizes the plasma membrane in muscle fibers, progressive weakness of skeletal, cardiac, and respiratory muscles is observed. First symptoms of abnormal gait and walking are observed in the affected boys between 3 and 5 years of age and by age 12, most boys are wheelchair-dependent [2]. Due to continuous degradation and asynchronous regeneration of the myofibers, the normal muscle tissue is replaced by fat deposits and scarring, as a result of chronic inflammation and fibrosis [3, 4]. Over time, the majority of DMD patients develop dilated cardiomyopathy, which is accompanied by deterioration of respiratory function leading to significant morbidity and premature death around the age of 20 due to the cardiac and respiratory failure. Despite significant scientific efforts, there is no cure for DMD patients. Currently, the available treatment options addressing cardiac and pulmonary problems include corticosteroids, vasodilators, antioxidants and supportive measures such as respiratory muscle training and non-invasive respiratory support [4–8]. Since the supportive measures and clinical management of DMD have not improved or halted progression of the disease, new therapeutic approaches are needed in order to restore the cardiac, respiratory, and skeletal muscles function to clinically relevant levels [9–11]. Different approaches targeting the genetic defect have been investigated including exon skipping therapy [4, 12], gene editing via viral vectors [13–16] and genome editing using CRISPR technology [14, 16]. Since gene therapies are usually targeting very specific mutations, they are not applicable to all DMD patients [17, 18]. Although the preliminary results for genetic correction are encouraging, the clinical efficacy is still debatable due to the safety concerns for the “off-target mutations”, tumorigenicity and sensitization [15, 19, 20]. Recent reports on the microdystrophin AVV-based therapy are promising, however side effects related to the dose-dependent immune response are of concern [21]. Cell-based therapies including myoblasts or mesoangioblasts transplantation, aiming for regeneration of the damaged muscles, have shown early promising results [22–25]. However, the reported limitations included the short-term cell engraftment, allogeneic immune response, and the side effects of the supportive immunosuppressive therapy [3, 26, 27]. Therefore, the optimal cell-based therapy should be characterized by low immunogenic phenotype, accessibility, proper expansion, and high transplanted cell survival rate which is crucial for the maintenance of the long-term engraftment without the need for immunosuppression [17, 22, 28–34]. We have addressed these specific unmet needs for cell-based therapies using a new chimerism-based tolerance inducing strategy for bone marrow and vascularized composite allotransplantation (VCA) and generated new chimeric cell lines of hematopoietic, mesenchymal and myoblast lineage origin for DMD therapy [35–39].

Moreover, we have applied the chimeric cell therapy concept to muscular dystrophy and created Dystrophin Expressing Chimeric (DEC) Human cells by fusion of human myoblasts (MB) derived from normal and DMD-affected donors (MB^N^/MB^DMD^). Administration of DEC therapy to the *mdx* mouse models of DMD resulted in restoration of dystrophin expression which correlated with significant improvement of cardiac, respiratory and skeletal muscle function [39, 40].

Furthermore, we have investigated the systemic effect of DEC therapy and confirmed restoration of dystrophin expression and reduced *mdx* muscle pathology which correlated with improvement of cardiac, pulmonary and skeletal muscle function at 90 days after systemic-intraosseous DEC administration [41]. Currently, the major challenge, precluding routine application of cell-based therapies in DMD is the lack of maintenance of long-term engraftment and function.

Therefore, considering the dynamic progression of DMD and to bring DEC technology closer to the clinical application, in the current study we have assessed a long-term (180 days) efficacy of human DEC therapy after systemic-intraosseous administration. Our goal was to assess the protective effect of DEC therapy in the most affected by the DMD organs before severe progression of the disease and before development of the overt DMD -related cardiomyopathy or pulmonary failure, to make it more clinically relevant, since once organ failure occurs, treatment with stem-cell based therapies would not reverse damage created by DMD disease.

Thus, in this study, the maintenance of long-term engraftment of DEC correlating with restoration of function was confirmed by normalized muscle morphology, reduced pathology, and improved function in the most severely affected by DMD organs including heart, diaphragm, and long skeletal muscles. This encouraging preclinical data introduces human DEC as a novel Advanced Therapy Medicinal Product (ATMP) with the potential to improve or halt progression of the disease and enhance quality of life of DMD patients.

## Materials and Methods

### Mice and Animal Care

This study was approved by the Institutional Animal Care and Use Committee (IACUC) of University of Illinois at Chicago, which is approved by the American Association for the Accreditation of Laboratory Animal Care (AAALAC). All animals received humane care in compliance with the ‘Principles of Laboratory Animal Care’ formulated by the National Society for Medical Research and the ‘Guide for the Care and Use of Laboratory Animal Resources’. Six- to eight-week-old male *mdx/scid* mice—the animal model for Duchenne Muscular Dystrophy (B10ScSn.Cg-*Prkdc*^*scid*^* Dmd*^*mdx*^/J, stock number 018018) with respective background wild type mice (C57BL/10ScSnJ, stock number 000476) were purchased from Jackson Laboratories. Animals were kept in a pathogen-free environment on a light/dark cycle. Prior to study initiation, aged matched male *mdx/scid* mice were ear tagged and randomized into following experimental groups: vehicle control injected with 60 µl phosphate-buffered saline, PBS (*n* = 4), DEC therapy group injected with 1 × 10^6^ DEC cells suspended in 60 µl PBS (*n* = 5), and DEC therapy group injected with 5 × 10^6^ DEC cells suspended in 60 µl PBS (*n* = 5).

### Cell Culture

Normal human myoblasts (MB) were purchased from Lonza Bioscience (Mapleton, IL, USA), and DMD-affected myoblasts were purchased from Creative Bioarray Ltd. (Shirley, NY, USA). Myoblasts (MB) were cultured in Skeletal Muscle Cell Growth Medium-2 (Lonza Clonetics, Mapleton, IL, USA) supplemented with the human Epidermal Growth Factor (hEGF); Fetal Bovine Serum (FBS); Dexamethasone; Gentamicin/Amphotericin B (GA) (Lonza Clonetics, Mapleton, IL, USA). Upon reaching 60–70% confluence, myoblasts were harvested using 0.25% trypsin/EDTA (Sigma-Aldrich, MO, USA). Enzymatic activity was inhibited with 10% serum-supplemented culture media. Human MBs were harvested between passages 3–7, which is optimal for the ex vivo cell fusion procedure.

### Cell Fusion Procedure

After harvesting, staining and viability assessment with 0.4% Trypan Blue (Gibco-ThermoFischer, Waltham, MA, USA), parent myoblasts (MB^N^ and MB^DMD^) were washed in serum-free media supplemented with antibiotics (1% Antibiotic–Antimycotic solution, Gibco-ThermoFischer, Waltham, MA, USA). Next cell fusion procedure was performed. As previously described [38–40] parent myoblasts (MB^N^ and MB^DMD^) were fluorescently labeled using PKH26 or PKH67 (Sigma-Aldrich, St. Louis, MO) membrane dyes respectively, according to manufacturer’s instructions. Parent cells were mixed, washed and fusion was performed using 1.46 g/mL PEG solution (PEG 4000, EMD) containing 16% DMSO (Sigma, St. Louis, MO) [42–44]. Fused cells were transferred to fluorescently activated cells sorting (FACS) buffers containing 5% HEPES, 1% EDTA and 5% FBS. Finally, cells presenting double (PKH26/PKH67) staining were selected via FACS (MoFlow Astrios, Beckman Coulter, San Jose, CA, USA) sorter and were used for intraosseous administration to the *mdx/scid* mice.

### Systemic–Intraosseous DEC Administration

Mice were anesthetized with 2% isoflurane inhalation along with 1 mL/kg buprenorphine subcutaneous injection. DEC intraosseous administration was performed as previously reported [39, 41]. Briefly, a 5 mm incision was made at the lateral-mid thigh level and muscles were separated to expose femoral bone. DEC cells were transferred in a 60 µl volume of sterile PBS to a tuberculin syringe (cat. ThermoFischer, Waltham, MA, USA). A 25G needle was used to aspirate 60 µl of bone marrow followed by DEC cells injection directly into the femur. Bone wax was applied to the injection site. Next, muscles were approximated, and the wound was closed with 5–0 nylon sutures. Animals recovered in a heated environment with post-operative monitoring and returned to the colony.

### Immunofluorescence for Dystrophin Expression Assessment

For immunofluorescence the optimal cutting temperature (OCT) frozen sections of the heart, diaphragm and gastrocnemius muscle were fixed with ice-cold acetone for 10 min and blocked for the unspecific binding with 10% normal goat serum for 60 min in 4 °C. Next, specimens were incubated with mouse monoclonal anti-human dystrophin (1:50, cat. ab15277, Abcam, Cambridge, UK) and mouse monoclonal anti-human spectrin (Leica, Clone RBC2/3D5, Biosystems, NCL-SPEC1) primary antibodies, followed by incubation with a goat anti-mouse conjugated AlexaFluor-647 secondary antibody for dystrophin (1:400, cat. A-21241, ThermoFischer, Waltham, MA, USA) and goat anti-mouse secondary antibody for spectrin (IgG) Alexa Fluor® 647 (1:400, ab150115, Abcam, Cambridge, UK). Appropriate positive and negative controls were used. Nuclear counter-staining was performed using 4′,6-diamidino-2-phenylindole (DAPI) (cat. ab104139, Abcam, Cambridge, UK). A Zeiss Meta confocal microscope with ZEN software (Carl Zeiss, Oberkochen, Germany) and Leica DM4000B microscope were used for fluorescence signal detection and analysis.

### Histology for Muscle Pathology Assessment

For histological analysis, the heart, diaphragm and gastrocnemius muscles were harvested, fixed in 10% neutral buffered formalin and embedded in paraffin. Next, the paraffin blocks were cut into 5 µm non-consecutive transverse cross-sections. Samples were deparaffinized and subsequently stained with appropriate staining kits, then mounted (Poly-Mount, PolySciences Inc. Warrington, PA, USA) to analyze overall muscle morphology, to assess infiltration of inflammatory cells, and to quantify the centrally nucleated fibers (CNF), fibers minimal Feret’s diameter size, and finally to assess the level of fibrosis. All histological assessments were done on 12 ROI (region of interest) acquired from three non-serial cross sections from n = 3 mice (12 ROI/organ/mouse or 36 ROI/organ/group) using ImageJ NIH software. The digital images were acquired (BX51/IX70 Olympus, Japan) and processed using ImageJ.

H&E-stained sections of target organs of heart, diaphragm and gastrocnemius muscle of DEC injected *mdx/scid* mice were assessed and compared with the vehicle-injected controls and Wild Type (WT) mice. Inflammation was assessed by counting the total number of small (5–9 inflammatory cells) and large (groups of 10 or more inflammatory cells) foci in the representative images. The numbers of foci counts were normalized to the area unit (number of foci/mm^2^).

For analysis of centrally nucleated fibers, H&E-stained sections of diaphragm and gastrocnemius muscle were assessed by counting the number of fibers with centrally located nuclei, which represent pathological changes in the muscle tissue. The number of CNF was compared to the total number of fibers and expressed as a percentage.

Fiber diameters were assessed by minimal Feret’s diameter on H&E-stained sections of heart, diaphragm, and gastrocnemius using ImageJ measurement plug-ins. Average number of fibers falling into 5 µm increments was normalized to the total number of fibers and expressed as a percentage.

For analysis of cardiac muscle fibrosis, cross sections of heart samples were stained with Picro-Sirius Red kit specific for cardiac muscle (Abcam, cat. 245,887, Cambridge, MA, USA). Fibrosis of the diaphragm and gastrocnemius muscle was analyzed in the sections, which were stained with Trichrome Stain kit (Abcam, cat. ab150686, Cambridge, MA, USA) for visualization of collagenous fibrotic tissue.

Pixels corresponding to the area stained in red (for Picro-Sirius), or blue (for Trichrome) or indicating collagenous areas reflecting fibrosis, were normalized to the total pixel area of the tissue in the assessed image. Results were expressed as a percentage of collagenous area versus total tissue area in the region of interest.

### Echocardiography for Cardiac Function Assessment

Echocardiography was performed as previously described [39, 41]. Briefly, mice were anesthetized with isoflurane (1–3%) and placed on the ultrasound stage. Paws were taped to the electrocardiograph (ECG) electrodes to monitor heart rate (maintained at 350–450 bmp). Assessments were made at the baseline—before administration of DEC and at 30 days, 90 days, and at 180 days endpoint after intraosseous DEC administration. M-mode echocardiographic images were obtained from the parasternal long axis view through the center of the left ventricle (FujiFilm, VisualSonics, Vevo 2100, Toronto, Canada) [45, 46]. Recordings were analyzed using VevoLab (ver. 3.2.0).

### Plethysmography for Pulmonary Function Assessment

Whole body plethysmography was applied for assessment of respiratory function as described elsewhere [41, 47]. Small animal plethysmography set-up (Buxco/DSI, St. Paul MN, USA) using FinePointe (Buxco/DSI) was applied. The assessment was performed at the study endpoint of 180 days post-DEC administration. The following parameters were calculated: Enhanced pause (Penh) and Expiration time (Te).

### Aurora Test for ex vivo Muscle Force Assessment

After animal euthanasia at the study endpoint of 180 days post-DEC administration, the contractile and passive properties of the gastrocnemius muscle (GM) were measured ex vivo using Aurora Scientific test system as described previously [38]. After dissection of the whole left and right GM, the Achilles tendon and proximal pole of muscle were attached to the force transducer. Muscle force was measured after establishing optimal length through a standardized stimuli pattern until reaching the maximal wave and maximal strain. Results were averaged for the right and left gastrocnemius muscles and reported as the force/muscle weight (g/g).

### Grip Strength Assessment

The assessment of *mdx/scid* mice motor function after systemic-intraosseous administration of DEC cells was tested by the right hindlimb grip strength test. The function was monitored up to a 180-day endpoint. The tests were performed weekly, for 26 weeks of follow-up. The order of animals for test performance was randomly assigned. A grip meter (Digital Force Gauge, HL-50) was used to measure the GM-specific force. This test allows forelimb force measurements, providing information on the muscle strength. The hook of a grip meter was placed touching the animal’s toes. Once gripped, it was repeatedly pulled 10 times, and the average maximum peak was used for further analysis.

### Statistical Analysis

Data are expressed as mean ± SEM (standard error of the mean). GraphPad Prism (ver. 9.2.1) software was used to perform statistical analysis. Two-tailed Student t-test for group comparisons was used to define statistical significance. Results were considered statistically significant for p < 0.05. The graphs represent mean values with standard error of mean (SEM), statistical significance is marked with asterisks: *p < 0.05, **p < 0.01, ***p < 0.001, ****p < 0.0001.

## Results

### I. The Long-Term Amelioration of the mdx Pathology in Cardiac, Respiratory and Skeletal Muscles at 180 days after Systemic-Intraosseous Administration of Human DEC Therapy to the mdx/scid Mouse Model of DMD.

This study assessed the long-term (180 days) effect of DEC therapy after systemic-intraosseous administration to the *mdx/scid* mice. At the study baseline, the six to eight-weeks old *mdx/scid* mice were randomly assigned to two therapy groups of 1 × 10^6^ and 5 × 10^6^ dose of human DEC cells in the respective groups. Following intraosseous administration of DEC, animals were monitored up to the study endpoint of 180 days, when they were assessed for muscle pathology and by the ex vivo and in vivo functional tests.

All animals tolerated well the intraosseous administration of both dosages of human DEC therapy into the femoral bone. The injections were performed under surgical microscope magnification of 20X and the PBS-based DEC cell solution was injected without any spill or leakage. There was no presence of hematoma, inflammation, or infection observed at the surgical site after injection and no side effects were observed during the entire follow-up period up to 180 days.

### Human DEC Therapy Increases Dystrophin Expression in Cardiac, Respiratory and Skeletal Muscles at 180 Days after Systemic Administration

**Fig. 1:**
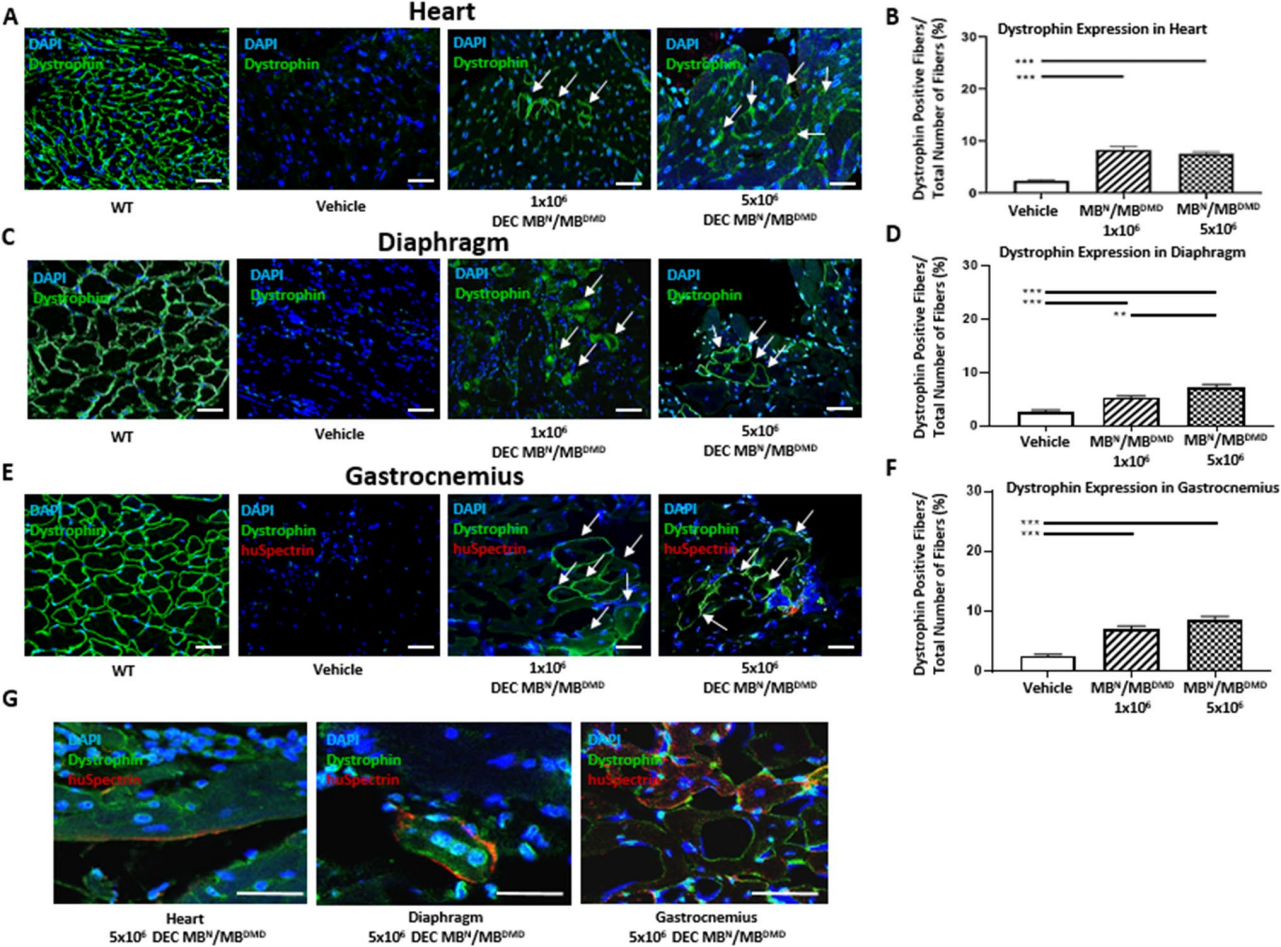
Systemic-intraosseous administration of human DEC (1 × 10^6^ and 5 × 10^6^) cells confirms long-term DEC engraftment and increased dystrophin expression in the heart, diaphragm and gastrocnemius muscle at 180 days after DEC transplant to the *mdx/scid* mice. Immunofluorescence images of dystrophin expression in the heart **(A)**, diaphragm **(C)** and gastrocnemius muscle (**E)** after systemic DEC transplant compared to vehicle and WT controls. White arrows indicate positive fibers, showing differences between the therapy groups and the vehicle control; Magnification 25X, scale bar = 50 μm; for merge: dystrophin (green), human spectrin (red), DAPI nuclei counterstaining (blue). **(G)** Representative immunofluorescence images of human spectrin (red) expression in the heart, diaphragm and gastrocnemius muscle samples of the *mdx/scid* hosts injected with human DEC (1 × 10^6^ and 5 × 10^6^), dystrophin (green), nuclei counterstained with DAPI (blue); Magnification 140X, scale bar 20 μm (ZEISS 710 META, Oberkochen, Germany). **(B, D, F)** Significant increase of dystrophin expression in target organs of: heart **(B)**, diaphragm—dose-dependent effect **(D)**, and gastrocnemius muscle **(F)** of DEC-injected compared to vehicle injected *mdx/scid* mice, n = 3/group, 10 ROI/organ/mouse. Dystrophin-positive fibers were counted and normalized to the total number of fibers. All data presented as mean ± SEM. One-way ANOVA with post-hoc Tukey’s test. **p < 0.01, *** *p* < 0.001

We previously reported restoration of dystrophin expression which correlated with short-term protection of cardiac and skeletal muscle function after systemic-intraosseous administration of murine and human DEC cells to the *mdx* mice [39, 41]. In the current study, to test the therapeutic and clinical potential of human DEC cell line we tested functional efficacy of long-term engraftment and dose–response of two doses of human DEC cells (1 × 10^6^ or 5 × 10^6^). Immunofluorescence (IF) staining confirmed long-term (180 days) engraftment and increased dystrophin expression in the selected target organs of heart, diaphragm and gastrocnemius muscle (GM) of *mdx/scid*-injected mice. The IF images of heart sections of both DEC therapy groups (Fig. 1A), revealed long-term significant increase in dystrophin expression in 1 × 10^6^ (8.27% ± 0.65%) and 5 × 10^6^ (7.52% ± 0.35%) DEC doses when compared to the vehicle-injected controls (2.32% ± 0.21%) (Fig. 1B).

The diaphragm IF sections (Fig. 1C) confirmed significant increase in dystrophin expression in both DEC-injected groups of 1 × 10^6^ (5.34% ± 0.36%) and 5 × 10^6^ (7.29% ± 0.52%) DEC cells when compared to the vehicle-injected controls (2.68% ± 0.36%). Moreover, a significant increase in dystrophin positive fibers was observed in 5 × 10^6^ dose when compared to 1 × 10^6^ DEC indicating a dose-dependent effect of DEC therapy on restoration of dystrophin in the diaphragm of *mdx/scid* mice (Fig. 1D)*.*

Assessment of gastrocnemius muscle IF sections (Fig. 1E) revealed increase of dystrophin expression in both DEC-injected groups of 1 × 10^6^ (7.09% ± 0.46%) and 5 × 10^6^ (8.67% ± 0.50%) DEC cells when compared to the vehicle-injected controls (2.57% ± 0.24%) (Fig. 1F). In addition, co-localization of dystrophin expression with expression of the human-specific spectrin (red) in the heart, diaphragm and GM samples of the *mdx/scid* hosts injected with both doses (1 × 10^6^ and 5 × 10^6^) of human DEC cells confirms the long-term engraftment of DEC and human origin of the dystrophin positive muscle fibers (Fig. 1G).

### DEC Therapy Reduces Inflammation in the DMD-Targeted Organs of Heart, Diaphragm, and GM at 180 days after Intraosseous Administration

We have previously confirmed short-term reduction of the inflammatory response at 90 days after systemic DEC administration [41]. Since inflammation is the hallmark of DMD progression, thus in this study, we assessed the long-term effect of DEC therapy on amelioration of inflammation in the DMD affected organs of heart, diaphragm and skeletal muscle.

**Fig. 2:**
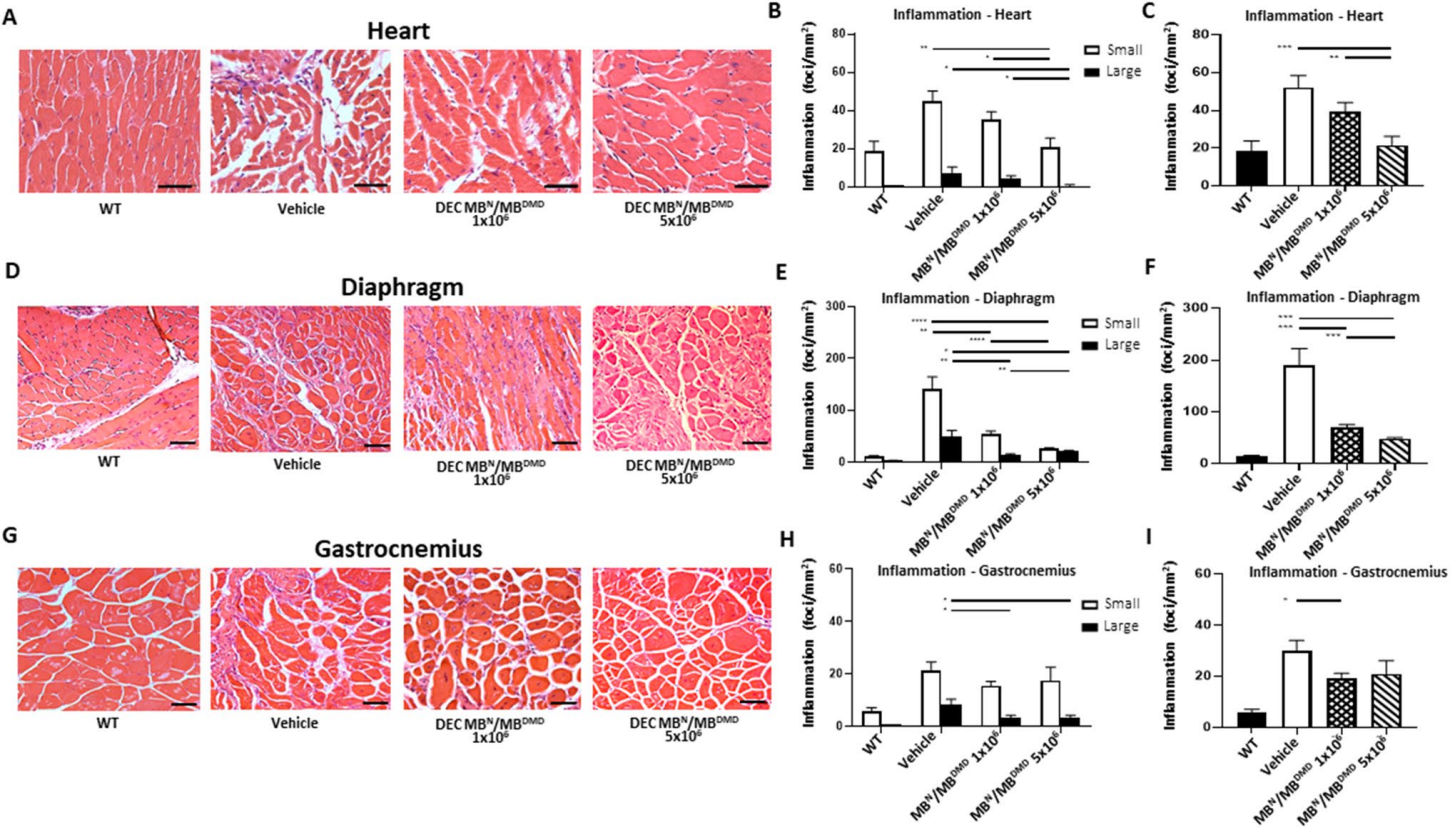
Administration of human DEC therapy (1 × 10^6^ and 5 × 10^6^) results in reduced inflammation in the heart, diaphragm and gastrocnemius muscle at 180 days following DEC administration. Representative images of Hematoxylin and Eosin (H&E) stained cross-sections of heart **(A)**, diaphragm **(D)**, and gastrocnemius muscle **(G)** confirmed reduced muscle inflammation in both DEC groups compared to vehicle-injected controls. Magnification 40x (heart panel) 20x (diaphragm and gastrocnemius muscle), scale bar 50 μm, n = 3/group, 12 ROI/organ/mouse. **(B)** In heart H&E sections the number of small and large foci was significantly reduced in both DEC groups and was dose-dependent. No large foci were seen in the 5 × 10^6^ group. **(C)** Dose-dependent reduction of total number of inflammatory foci was observed in cardiac muscle in both DEC groups but not in vehicle-injected group and was dose-dependent. **(E)** In diaphragm, significant dose-dependent reduction of small and large inflammatory foci was observed in DEC-injected compared to vehicle-injected mice. **(F)** Total number of inflammatory foci in diaphragm was significantly reduced in both DEC groups when compared to vehicle-injected controls and was dose-dependent. **(H)** In gastrocnemius muscle significant reduction of the total number of inflammatory foci was observed in both DEC-injected mice when compared to vehicle-injected controls. **(I)** Total number of inflammatory foci in GM was reduced in DEC-injected compared to vehicle-injected groups. Data presented as mean ± SEM. One-way ANOVA with post-hoc Tukey’s test *p < 0.05, **p < 0.01, *** p < 0.001, **** p < 0.0001

Histological assessment of the number of small, large and total inflammatory foci (combined small and large) in the H&E sections of heart, diaphragm and gastrocnemius muscle confirmed reduced inflammation at 180 days after intraosseous administration of human DEC (1 × 10^6^ and 5 × 10^6^) cells to the *mdx/scid* mouse. In the cardiac muscle sections (Fig. 2A) the number of small and large inflammatory foci was reduced after administration of both doses (1 × 10^6^ and 5 × 10^6^) of human DEC. The number of small inflammatory foci was significantly decreased after administration of 5 × 10^6^ of human DEC (21.05 ± 4.57 foci/mm^2^) compared to the vehicle-injected controls (44.80 ± 5.48 foci/mm^2^). There was a dose-dependent reduction of inflammatory foci observed in higher DEC dose of 5 × 10^6^ (21.05 ± 4.57 foci/mm^2^) compared to 1 × 10^6^ (35.25 ± 4.04 foci/mm^2^) dose. Interestingly, there were no large inflammatory foci observed in heart samples of mice injected with 5 × 10^6^ DEC (0.00 ± 0.00) compared to 1 × 10^6^ dose (4.25 ± 1.56 foci/mm^2^) and vehicle-injected controls (7.45 ± 2.96 foci/mm^2^) (Fig. 2B). Assessment of the total number of inflammatory foci revealed a significant and dose-dependent decrease in the number of inflammatory foci in heart sections of DEC-injected mice with 1 × 10^6^ (39.50 ± 4.74 foci/mm^2^) and the 5 × 10^6^ (21.64 ± 4.68 foci/mm^2^) cells when compared to the vehicle-injected controls (52.25 ± 6.20 foci/mm^2^) (Fig. 2C). In the diaphragm sections (Fig. 2D) significantly reduced number of small inflammatory foci was observed in mice injected with both DEC doses of 1 × 10^6^ (55.44 ± 5.18 foci/mm^2^) and 5 × 10^6^ (26.72 ± 1.48 foci/mm^2^) when compared to the vehicle-injected controls (140.60 ± 23.92 foci/mm^2^). Analysis of large foci counts revealed a dose-dependent reduction in the number of inflammatory foci after both DEC doses of 1 × 10^6^ (15.00 ± 2.21 foci/mm^2^) and 5 × 10^6^ (22.14 ± 1.08 foci/mm^2^) when compared to the vehicle-injected controls (50.02 ± 11.90 foci/mm^2^) (Fig. 2E). Assessment of total number of inflammatory foci revealed a significant decrease of foci number in diaphragm sections of *mdx/scid* mice injected with both DEC doses of 1 × 10^6^ (70.45 ± 4.83 foci/mm^2^) and 5 × 10^6^ DEC (48.86 ± 1.52 foci/mm^2^) compared to the vehicle-injected controls (190.60 ± 31.92 foci/mm^2^) (Fig. 2F). Analysis of gastrocnemius muscle (Fig. 2G) sections revealed reduced numbers of both, the small and large inflammatory foci in DEC-injected *mdx/scid* mice. Specifically, significantly reduced number of large inflammatory foci was observed in DEC dose of 5 × 10^6^ (3.45 ± 0.93 foci/mm^2^) compared to the vehicle injected controls (8.42 ± 2.01 foci/mm^2^) (Fig. 2H). Total number of inflammatory foci assessed in gastrocnemius muscle samples revealed a significant decrease in 1 × 10^6^ DEC dose (19.09 ± 2.14 foci/mm^2^) compared to vehicle-injected controls (29.88 ± 4.19 foci/mm^2^) (Fig. 2I).

### DEC Therapy Reduces Percentage of Centrally Nucleated Fibers in the Diaphragm and Gastrocnemius Muscle at 180 days after Systemic -Intraosseous Administration

**Fig. 3:**
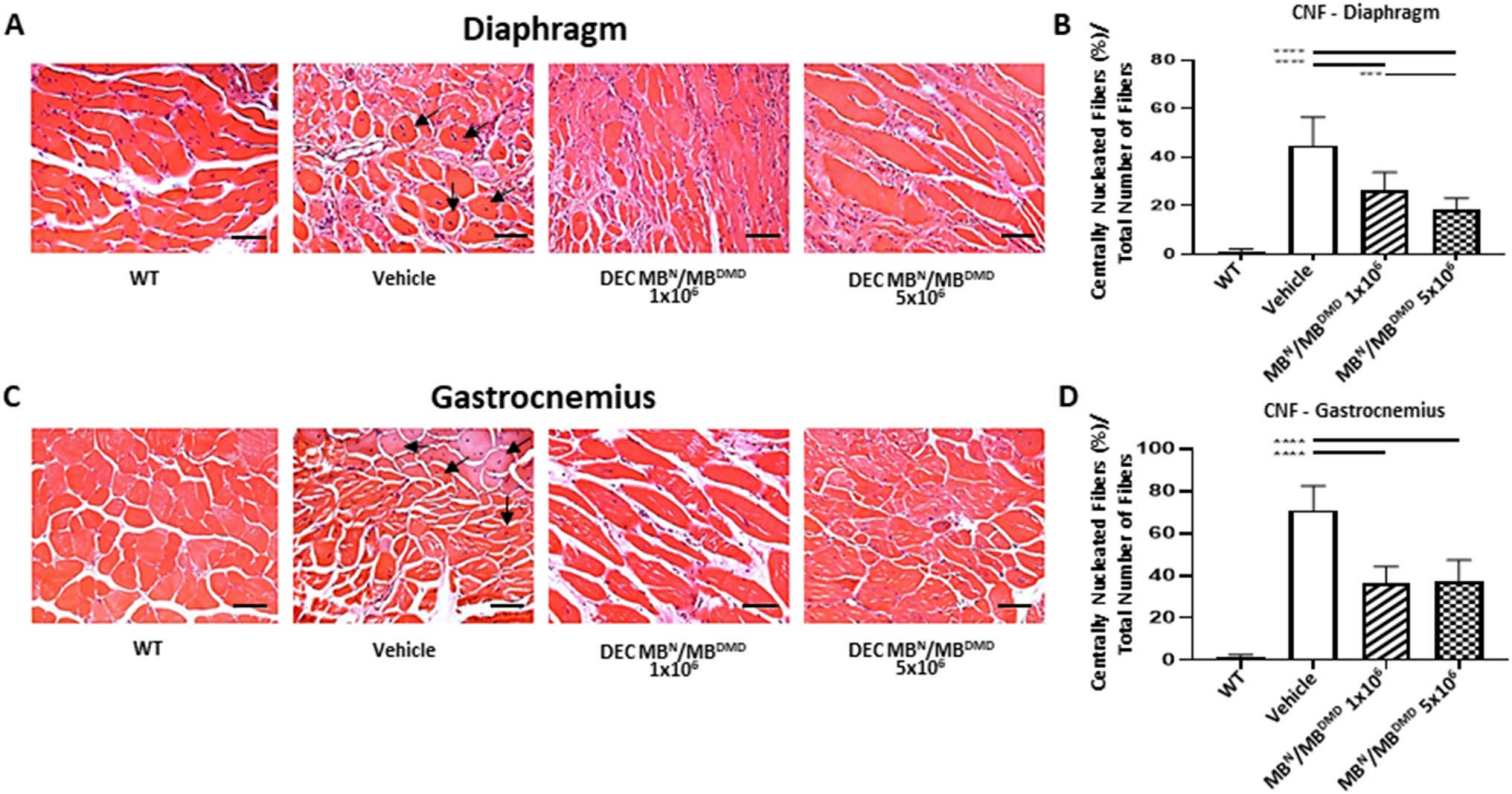
Systemic-intraosseous transplantation of human DEC (1 × 10^6^ and 5 × 10^6^) improves muscle pathology via reduced number of Centrally Nucleated Fibers (CNF) at 180 days after administration to *mdx/scid* mice. Representative images of Hematoxylin and Eosin **(**H&E) stained transverse sections of diaphragm **(A)** and gastrocnemius muscle **(C)** confirmed reduced number of centrally nucleated fibers (CNF) in DEC-injected groups compared to vehicle controls. Magnification 20x, scale bar 50 μm, *n* = 3/group, 12 ROI/organ/mouse. Significant dose-dependent reduction of CNF in diaphragm **(B)** and gastrocnemius muscle **(D)** in both DEC-injected, but not vehicle-injected mice. Number of CNF was normalized to the total number of fibers. CNF were marked with black arrows. Data presented as mean ± SEM. One-way ANOVA with post-hoc Tukey’s test. *** *p* < 0.001, **** *p *< 0.0001

The central location of cell nuclei is a hallmark of DMD disease and is characteristic for immature fibers, while mature muscle fibers have nuclei located peripherally. Thus, to further confirm potential beneficial effect of DEC therapy on reduction of muscle pathology, we assessed the number of centrally nucleated fibers (CNF), an indicator of *mdx* pathology in the diaphragm and gastrocnemius muscles at 180 days after systemic DEC administration. Histology specimens of diaphragm (Fig. 3A) confirmed significant decrease of CNF in *mdx/scid* mice injected with both doses of human DEC therapy 1 × 10^6^ DEC cells (26.15% ± 7.46%) and 5 × 10^6^ DEC cells (18.25% ± 4.81%), when compared to vehicle-injected controls (44.90% ± 11.41%). Furthermore, reduction of CNF fibers count was dose-dependent revealing significant drop in 5 × 10^6^ dose compared to 1 × 10^6^ DEC dose (Fig. 3B). Moreover, the protective effect of DEC therapy was confirmed in gastrocnemius muscle of DEC-injected *mdx/scid* mice (Fig. 3C), where a significant decrease of CNF was observed after administration of 1 × 10^6^ DEC (36.51% ± 7.52%) and 5 × 10^6^ DEC (37.51% ± 9.91%), when compared to the vehicle-injected controls (71.16% ± 11.38%) (Fig. 3D).

### Intraosseous Administration of Human DEC Therapy Normalizes Muscle Fiber Diameters in the DMD-Targeted Organs of mdx/scid mice at 180 days after DEC Transplant

Presence of variations in the muscle fiber diameter size represents characteristic features of muscles affected by DMD. We reported short term normalization of fiber size after DEC therapy [41]. Thus, in this study to further assess clinical applicability of DEC, we used Feret’s method to measure both, the overall change and the maintenance of normalization of fiber size diameters at the long-term (180 days) follow-up after systemic DEC administration.

**Fig. 4:**
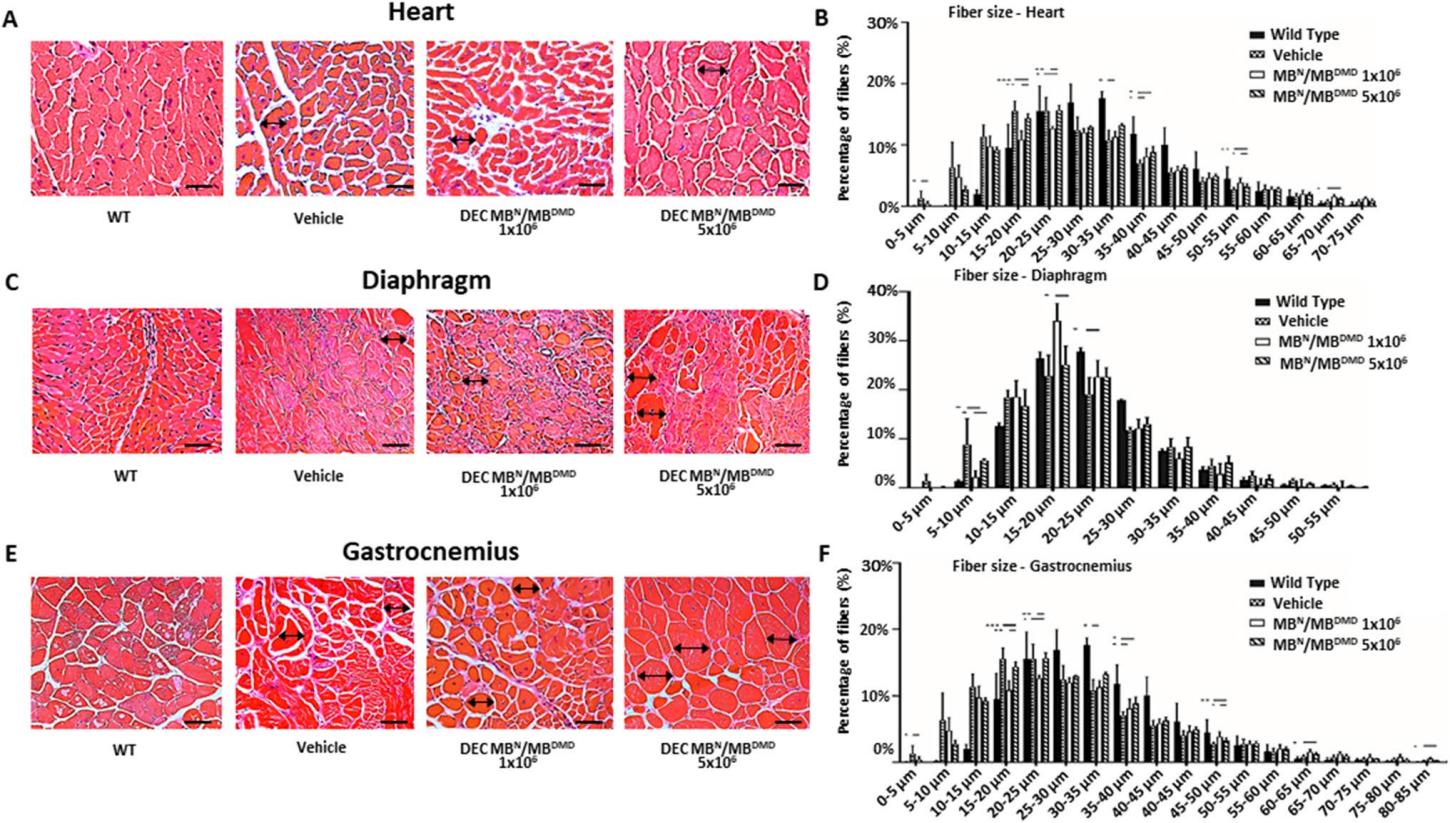
Human DEC (1 × 10^6^ and 5 × 10^6^) administration improves muscle morphology via normalized fiber size and homogeneity in the heart, diaphragm and gastrocnemius muscle at 180 days after systemic DEC administration. Representative histology images of Hematoxylin and Eosin (H&E) stained cross-sections of the cardiac **(A),** diaphragm **(C)** and gastrocnemius muscle **(E)** of *mdx/scid* mice at 180 days after systemic-intraosseous administration of DEC therapy compared with vehicle-injected and WT controls. Magnification 40x, scale bar 50 μm (heart); magnification 20x, scale bar 100 μm (diaphragm and gastrocnemius muscle), n = 3/group, 12 ROI/organ/mouse **(B)** Feret’s diameter measurements in the heart revealed a right-ward shift in fiber size distribution towards larger fibers in both DEC injected groups, bars represent means ± SEM. **(D)** Fiber size in the diaphragm was increased after DEC therapy when compared to vehicle controls, bars represent mean ± SEM. **(F)** In the gastrocnemius muscle there was a significant increase in the fiber size of DEC-injected compared to vehicle injected mice specifically in the fiber size increment between 30-35 µm. Fibers diameters were marked with black arrows. Data presented as mean ± SEM. Two sample t-test assuming unequal variances. *p < 0.05, **p < 0.01, *** *p* < 0.001, **** *p* < 0.0001

In the heart sections of the *mdx/scid* mice injected with both DEC doses, (Fig. 4A) a significant increase was observed in in the fiber size range within 20–25 µm (15.67% ± 4.86%) and 25–30 µm range (7.34% ± 2.89%) and was corresponding with the decrease in the number of smaller fibers within 10–15 µm range (33.41% ± 5.74%) when compared with the vehicle-injected controls (11.03% ± 1.2%, 3.43% ± 0.21%, and 39.31% ± 1.77%, respectively). This shift represents a normalization of fiber size approaching the wild-type phenotype (Fig. 4B). Histological assessment of diaphragm sections (Fig. 4C), revealed significant increase in fiber size diameters in the range of 15–20 µm (34.05% ± 3.61%) as well as 20–25 µm range (22.63% ± 3.40%) for both DEC doses when compared to the vehicle-injected controls. Interestingly, administration of higher DEC dose of 5 × 10^6^ cells, revealed increase in the third compartment of measured fiber diameters in the range of 25–30 µm, thus confirming dose dependent effect of DEC therapy on the maintenance of long-term normalization of fiber size diameters (22.79% ± 4.28%, and 19.08% ± 3.40% respectively),15–20 µm (25.12% ± 3.80%) range, 20–25 µm (22.63% ± 1.88%) range and 25–30 µm (12.99% ± 1.35%) (11.73% ± 0.59% at 25–30 µm range) (Fig. 4D). Similar findings were confirmed in the gastrocnemius muscle samples (Fig. 4E), where a shift in fiber size distribution towards larger fibers was observed in *mdx/scid* mice injected with both doses of DEC therapy: 5 × 10^6^ DEC (13.26% ± 0.27%) and 1 × 10^6^ DEC (12.04% ± 0.82%), when compared to the vehicle-injected controls (10.76% ± 1.70%). The presented data demonstrated a trend towards a dose-dependent response, where the higher DEC dose showed a stronger right-ward shift (towards increased mean fiber diameter) in the fiber size distribution over the lower DEC treatment dose (Fig. 4F).

### Systemic Administration of Human DEC Therapy Reduces Fibrosis in Cardiac, Respiratory and Skeletal Muscles at 180 days after DEC Transplant

Muscle fibrosis is the hallmark of muscular dystrophies and is progressing over the course of the disease. We have previously confirmed reduction of cardiac fibrosis in the *mdx* and *mdx/scid* mice models of DMD as well as amelioration of fibrosis in the diaphragm and gastrocnemius muscle at 90 days after systemic human DEC administration [39, 41]. In this study, the goal was to assess the long-term effect of DEC therapy on reduction of muscle fibrosis in the most severely DMD- affected organs of heart, diaphragm and gastrocnemius muscle.

**Fig. 5:**
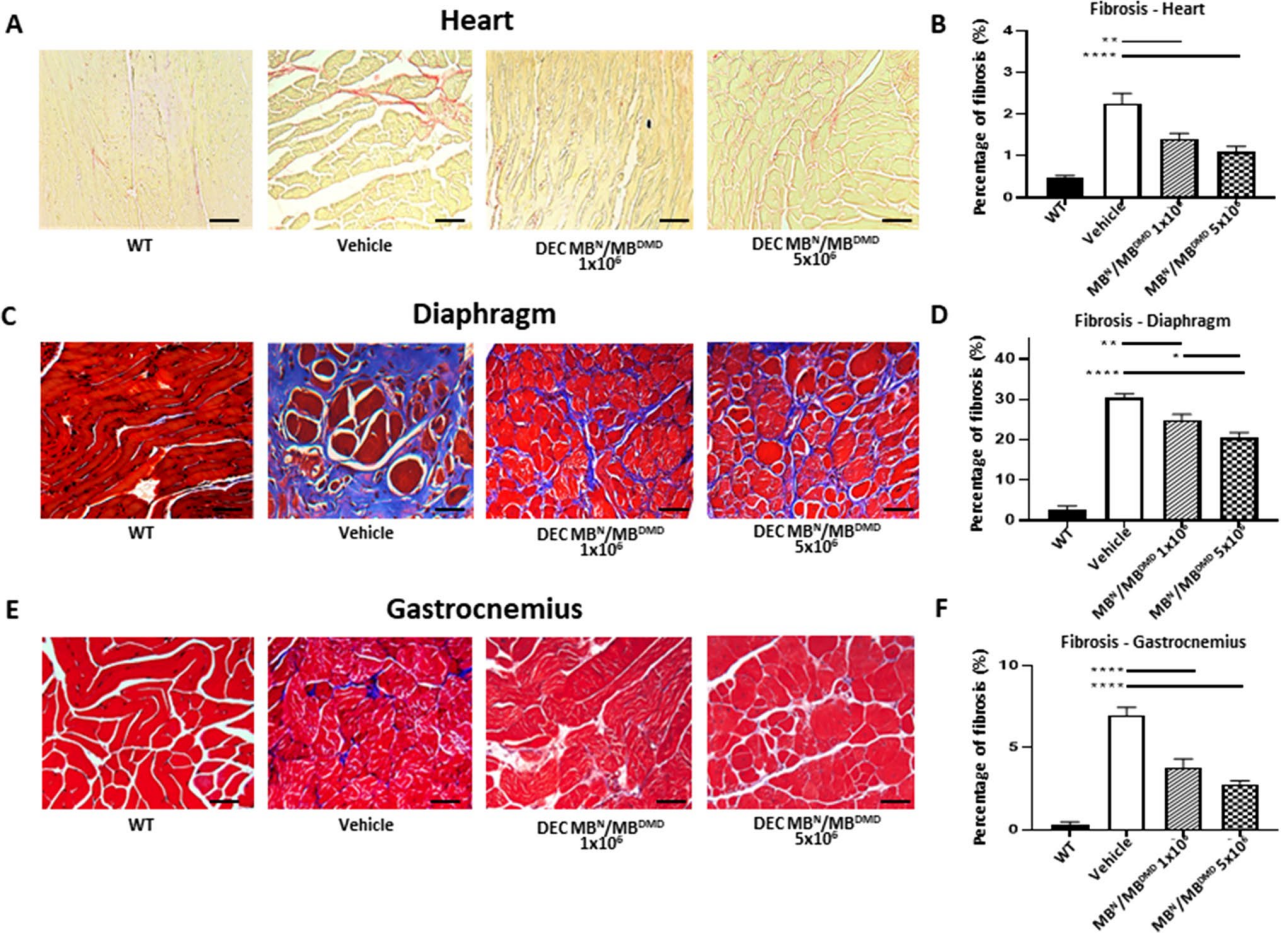
Intraosseous DEC (1 × 10^6^ and 5 × 10^6^) administration results in reduced fibrosis in the heart, diaphragm, and gastrocnemius muscle at 180 days after transplant. **(A)** Picro-Sirius staining of heart cross-sections assessing fibrosis after DEC therapy compared to vehicle and WT controls; fibrotic collagenous tissue (red), muscle fibers (yellow). Magnification 20x, scale bar = 50 µm, n = 3/group, 12 ROI/organ/mouse. **(B)** In the cardiac muscle, significant reduction of fibrosis was observed in both DEC-injected groups compared to vehicle-injected controls. Trichrome stained cross-sections of diaphragm **(C)** and gastrocnemius muscle **(E)** confirmed significantly reduced fibrosis in both DEC-injected mice compared to vehicle-injected controls. Blue-stained areas represent collagenous tissue (fibrosis) and red-stained areas represent muscle fibers. Magnification 20x, scale bars = 100 µm. **(D)** In the diaphragm sections, significant, dose-dependent reduction of fibrosis was observed in both DEC-injected but not vehicle- injected mice. **(F)** In gastrocnemius muscle, significant decrease of fibrosis was observed in both DEC-injected groups compared to vehicle-injected controls. Fibrosis was measured and normalized to total tissue area. Data presented as mean ± SEM. Two sample t-test assuming unequal variances. Abbreviations: *p < 0.05, **p < 0.01, *** *p* < 0.001, **** *p* < 0.0001

Assessment of fibrosis in the heart sections of *mdx/scid* mice by Picro-Sirius staining (Fig. 5A) revealed a significant reduction in the percentage of fibrotic changes after administration of both DEC doses of 1 × 10^6^ (1.41% ± 0.13%) and 5 × 10^6^ DEC cells (1.10% ± 0.12%), when compared to the vehicle-injected controls (2.26% ± 0.24%) (Fig. 5B). Assessment of diaphragm fibrosis by Trichrome Blue staining (Fig. 5C) confirmed significant decrease in the collagenous and fibrotic tissue deposits in the DEC-injected 1 × 10^6^ DEC cells (24.88% ± 1.43%) and 5 × 10^6^ DEC cells (20.66% ± 1.16%) when compared to the vehicle-injected (30.43% ± 0.93%) *mdx/scid* mice.

Moreover, this effect was dose-dependent showing lower percentage of fibrosis in the mice injected with higher dose of 5 × 10^6^ DEC cells (Fig. 5D). Furthermore, protective effect of DEC therapy was confirmed by reduction of fibrosis on the Trichrome stained gastrocnemius muscle samples (Fig. 5E) of mice injected with both DEC doses of 1 × 10^6^ DEC cells (3.81% ± 0.53%) and 5 × 10^6^ DEC cells (2.77% ± 0.21%) when compared to vehicle-injected *mdx/scid* mice (6.98% ± 0.48%) (Fig. 5F), thus, confirming amelioration of fibrosis at 180 days after systemic DEC administration.

### II. Long-term Functional Improvement of Cardiac, Respiratory and Skeletal Muscles at 180 Days after Systemic—Intraosseous Administration of Human DEC Therapy to the mdx/scid mouse model of DMD.

### Echocardiography Reveals Improvement of Cardiac Functional Parameters at 180 days after Human DEC Administration

Since progressive cardiomyopathy is responsible for premature death of DMD patients, evaluation of cardiac function is essential for assessment of disease severity and prognosis. We have reported the protective effect of DEC therapy on cardiac function in *mdx* and *mdx/scid* mouse models of DMD at 90 days after systemic DEC administration [39, 41]. In the current study, to check the potential clinical value of the systemic application of DEC therapy, we have assessed the dose-dependent effect of human DEC cells (1 × 10^6^ and 5 × 10^6^ dose) on cardiac function monitored by echocardiography over the entire follow-up period from the baseline over and at day 30, day 90 and day 180 after systemic-intraosseous injection of DEC.

**Fig. 6:**
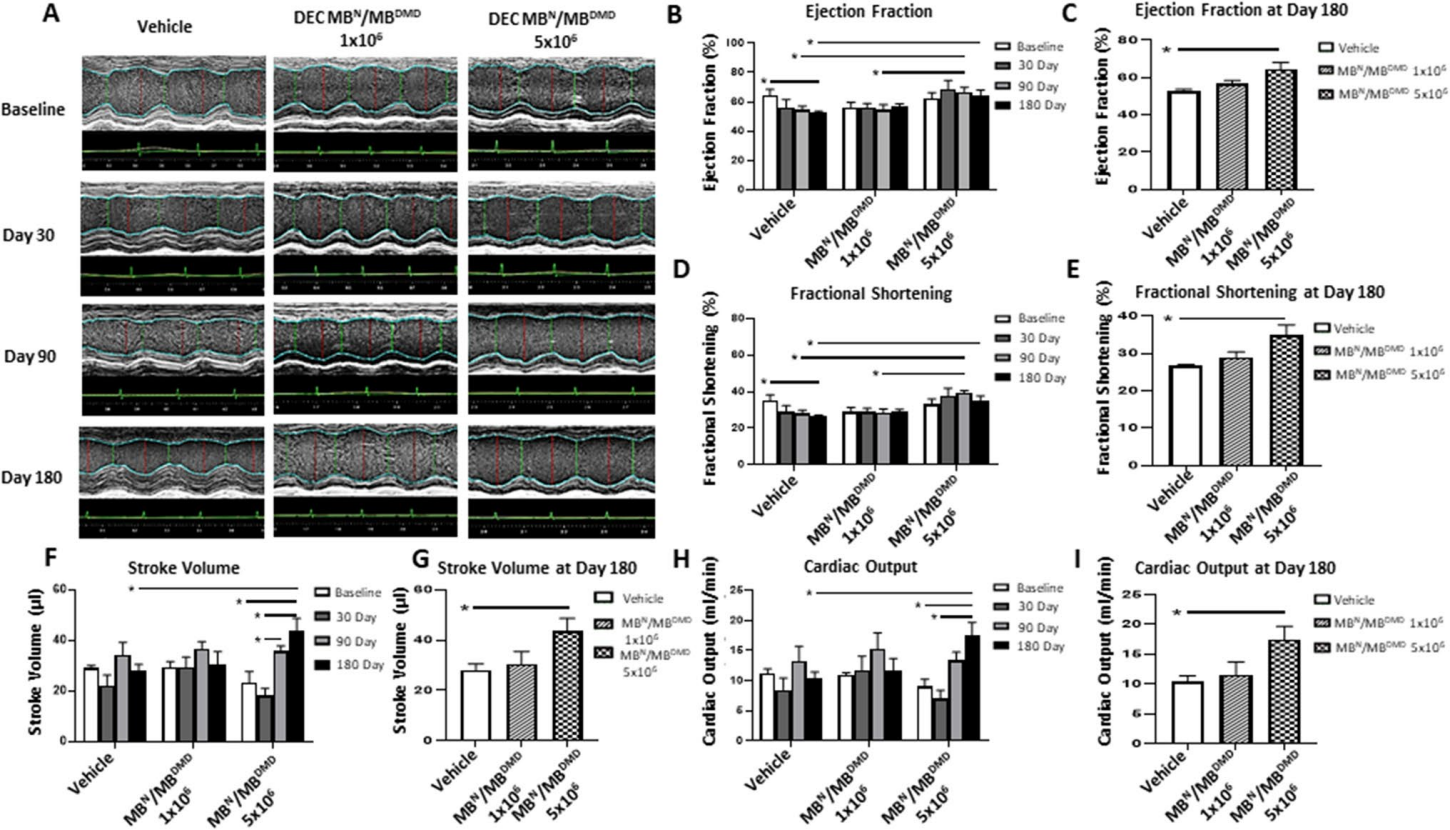
Administration of human DEC (1 × 10^6^ and 5 × 10^6^) results in long-term maintenance of cardiac function up to 180 days after systemic DEC transplant to the *mdx/scid* mice. **(A)** M-mode of the left ventricular (LV) parasternal long axis and LV tracing measurement after DEC administration compared to vehicle control. Vehicle-injected mice demonstrated gross thickening of the posterior LV wall and reduced size of the LV compared to DEC-injected *mdx/scid* mice. VEVO 2100 system (VisualSonics) and a 30-MHz cardiac probe (RMV707B), VEVO Lab ver. 3.2.0. **(B)** Echocardiography at 180 days post-transplant revealed maintenance of Ejection fraction (EF) after 1 × 10^6^ DEC dose and significant dose-dependent increase of EF after administration of 5 × 10^6^ DEC dose. In contrast, vehicle-injected mice revealed a significant drop in EF indicating progressive impairment of cardiac function. **(C)** At day 180 post-transplant, significant dose-dependent increased EF values were observed in the 5 × 10^6^ DEC group compared to vehicle-injected controls, confirming long-term protective effect of DEC therapy. **(D)** Fractional shortening (FS) in the 1 × 10^6^ DEC group was maintained at baseline values throughout 180 days follow-up, whereas significant dose-dependent increase in FS values was observed after administration of 5 × 10^6^ DEC dose. In contrast, vehicle-injected mice revealed a significant drop in FS. **(E)** At day 180 post-transplant significant, dose-dependent increase of FS was observed in the 5 × 10^6^ DEC injected mice confirming long-term protective effect of DEC therapy. **(F)** Stroke volume (SV) in the 1 × 10^6^ DEC group was maintained at baseline values throughout 180 days, whereas administration of higher DEC dose of 5 × 10^6^ DEC cells revealed continuous and significant increase in SV values from day 30 up to day 180. **(G)** At day 180 SV in the 5 × 10^6^ DEC-injected mice was significantly increased, confirming long-term and dose-dependent protective effect. **(H)** Cardiac output (CO) in the 1 × 10^6^ DEC group was maintained at the baseline values throughout 180 days follow-up whereas administration of higher DEC dose of 5 × 10^6^ DEC cells revealed continuous and significant increase in SV values from day 30 up to day 180. **(I)** At day 180 the CO in the 5 × 10^6^ DEC injected mice was significantly increased compared to vehicle-injected controls confirming long-term and dose-dependent protective effect. Data presented as mean ± SEM. Mann–Whitney test, *n* = 4. *p < 0.05

The long-axis brightness B-mode view through the center of the left ventricle was used to perform M-mode echocardiography for the assessment of morphometric parameters using the VEVO Lab software. M-mode imaging at 30-, 90- and 180-days post-transplant revealed gross thickening of the posterior left ventricular wall in the control mice and reduced left ventricular chamber size in DEC-injected *mdx/scid* mice (Fig. 6A). The images at 30, 90 and 180 days after intraosseous human DEC transplant confirm a protective effect of DEC therapy on the maintenance of LV function, and morphology in *mdx/scid* mice injected with two doses of human DEC cells. The morphometric parameters are summarized in Table 1 and include LVESD (left ventricular end systolic diameter), LVEDD (left ventricular end diastolic diameter), LVESV (left ventricular end systolic volume), LVEDV (left ventricular end diastolic volume). There were no significant differences observed in the morphometric parameters of DEC-treated versus vehicle injected mice as assessed by M-mode measurements by VevoLab software.

**Table 1:** Echocardiographic assessment of morphometric cardiac parameters at baseline and in response to DEC therapy at 30, 90, and 180 days after systemic-intraosseous administration of DEC therapy into *mdx/scid* mice. Morphometric parameters: LVESD = left ventricular end systolic diameter, LVEDD = left ventricular end diastolic diameter, LVESV = left ventricular end systolic volume; LVEDV = left ventricular end diastolic volume; There were no significant differences observed in the morphometric parameters of DEC-treated versus vehicle injected control mice as assessed by M-mode measurements by VevoLab software. All data presented as mean ± SEM, non-parametric, unpaired Student's t-test were performed

Morphometric parameters				
Experimental group/ Timepoint	LVESD (mm)	LVEDD (mm)	LVESV (μl)	LVEDV (μl)
Baseline				
Vehicle (*n* = 4)	2.30 ± 0.54	3.36 ± 0.29	17.71 ± 7.33	46.80 ± 9.26
MBN/MBDMD1 × 106 (*n* = 4)	2.50 ± 0.3	3.53 ± 0.39	23.99 ± 8.47	53.27 ± 12.49
MBN/MBDMD5 × 106 (*n* = 4)	2.01 ± 0.38	3.03 ± 0.39	18.91 ± 10.55	42.33 ± 6.67
Day 30				
Vehicle (*n* = 4)	2.25 ± 0.43	3.13 ± 0.47	18.88 ± 7.31	41.00 ± 12.60
MBN/MBDMD1 × 106 (*n* = 4)	2.62 ± 0.32	3.63 ± 0.37	25.84 ± 8.21	56.50 ± 13.65
MBN/MBDMD5 × 106 (*n* = 4)	2.66 ± 1.71	3.54 ± 2.69	10.84 ± 11.20	29.08 ± 16.71
Day 90				
Vehicle (*n *= 4)	2.79 ± 0.27	3.86 ± 0.14	30.86 ± 6.96	65.26 ± 5.89
MBN/MBDMD1 × 106 (*n* = 4)	2.83 ± 0.17	3.93 ± 0.13	31.17 ± 4.29	67.95 + 5.49
MBN/MBDMD5 × 106 (*n* = 4)	2.30 ± 0.34	3.60 ± 0.27	19.39 ± 7.06	55.33 ± 10.11
Day 180				
Vehicle (*n* = 4)	2.16 ± 0.28	3.36 ± 0.29	25.53 ± 6.04	53.69 ± 10.68
MBN/MBDMD1 × 106 (*n* = 4)	2.50 ± 0.34	3.53 ± 0.53	23.06 ± 6.85	53.45 ± 16.63
MBN/MBDMD5 × 106 (*n* = 4)	2.65 ± 0.28	3.92 ± 0.19	25.92 ± 7.00	69.61 ± 9.42

Assessment of Ejection Fraction (EF) in mice injected with 1 × 10^6^ DEC cells revealed an increase over the vehicle control EF and revealed maintenance of the EF values at baseline levels up to 180 days after DEC administration. Administration of higher DEC dose of 5 × 10^6^ resulted in significantly higher EF (66.86% ± 3.13%) at day 90 and at day 180 (64.40% ± 3.81%) compared with vehicle-injected controls (54.41% ± 2.82% and 52.86% ± 0.88%, respectively) (Fig. 6B). In addition, a gradual drop in ejection fraction which was observed in the vehicle-injected *mdx* mice, when compared to baseline (baseline – 64.29% ± 4.25%; day 30 – 56.16% ± 5.48%), and at day 180 a significant EF drop was observed (day 180 – 52.86% ± 0.88%) in the treated mice (Fig. 6C). This confirms a gradual progression of the DMD disease and decline of cardiac function over the 26-weeks follow-up period in the non-injected control mice.

Similarly, echocardiographic evaluation of Fractional Shortening (FS) confirmed maintenance of FS values at baseline levels for 1 × 10^6^ DEC dose over the entire 180 days follow-up, whereas administration of 5 × 10^6^ DEC resulted in a continuous increase in the FS values observed from the baseline until day 90 (baseline – 33.20% ± 2.79%; day 30—37.55 ± 4.32%; day 90 – 39.14 ± 1.47%). Additionally, at 90 days, mice injected with 5 × 10^6^ DEC dose demonstrated increased FS compared to the vehicle injected controls (28.05% ± 1.70%). The 1 × 10^6^ DEC dose revealed similar trends (Fig. 6D). At day 180, the FS values increased in 5 × 10^6^ DEC dose (34.83% ± 2.80%) compared with the vehicle-injected controls (26.57% ± 0.52%). In contrast, a gradual FS drop was only observed in the vehicle-injected controls over the entire follow-up period and at 180 days, revealed significant decrease when compared with baseline values (baseline – 35.13% ± 3.02%; day 180 – 26.57% ± 0.52) confirming progression of the DMD disease (Fig. 6E).

Assessment of Stroke Volume (SV) for 1 × 10^6^ DEC dose revealed maintenance of SV at the baseline levels (baseline – 29.27 ± 2.38; day 180 – 30.39 ± 5.16 µl) over the entire 180 days follow-up period. Administration of higher DEC dose (5 × 10^6^ cells) resulted in continuous increase in the SV values from day 30 up to day 180 (baseline – 23.42 ± 4.19 µl; day 30 – 18.23 ± 2.75 µl; day 90 – 35.94 ± 1.93 µl; day 180 – 43.69 ± 4.96) (Fig. 6F) and at 180 days, SV was significantly increased when compared to the vehicle-injected controls (28.15 ± 2.37 µl) and 1 × 10^6^ DEC dose (30.39 ± 5.16 µl) confirming the long-term dose-dependent protective effect of DEC therapy on cardiac function (Fig. 6G).

Evaluation of Cardiac Output (CO) after administration of 1 × 10^6^ DEC cells demonstrated maintenance of CO values at baseline levels over the entire 180 days period (Fig. 6H). Administration of higher DEC dose of 5 × 10^6^ revealed continuous increase of CO values from day 30 to day 180 (baseline – 9.05 ± 2.34 ml/min; day 30 – 7.07 ± 2.66 ml/min; day 90 – 13.43 ± 2.65 ml/min; day 180 – 17.46 ± 3.92) and at day 180, was significantly increased compared to vehicle-injected controls (10.38 ± 1.07 ml/min) and 1 × 10^6^ DEC dose (10.38 ± 2.15 ml/min) confirming the long-term, dose-dependent protective effect of DEC therapy on cardiac function (Fig. 6I).

### Human DEC Therapy Improves Respiratory Function at 180 days after Systemic-Intraosseous Administration

Since pulmonary fibrosis and respiratory failure is significantly contributing to the premature death of DMD patients, we evaluated pulmonary function via plethysmography at the baseline and at 180 days after systemic administration of two doses (1 × 10^6^ and 5 × 10^6^) of DEC cells.

**Fig. 7:**
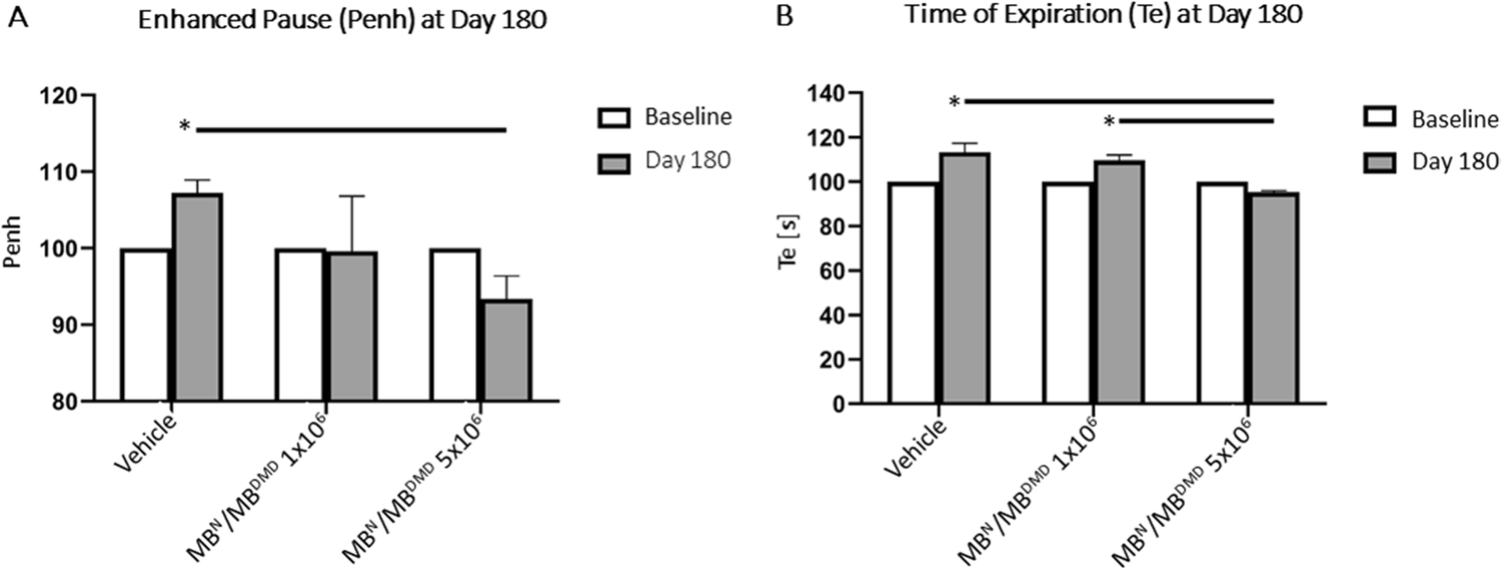
Systemic-intraosseous administration of human DEC results in reduced *mdx*-mediated respiratory disease at 180 days after transplant. Assessment of respiratory function by whole body plethysmography revealed: **(A)** significant decrease in enhanced pause (PENH) in 5 × 10^6^ DEC-injected mice when compared to the vehicle-injected control at 180 days after DEC transplant **(B)** Significant decrease in expiration time (Te) was observed in both DEC-injected groups compared to the vehicle-injected controls and was dose dependent, confirming protective effect of DEC therapy on pulmonary function. Data were normalized to individual animals' Penh and Te values at baseline (starting at 100% at a baseline for all of the groups, as indicated by the white bars). Data presented as mean ± SEM; Two sample t-test assuming unequal variances. Buxco Instrument. Abbreviations: *p* < 0.05

Measurement of respiratory function at 180 days after systemic DEC administration confirmed reduced *mdx*-mediated respiratory disease confirmed by a drop in the enhanced pause (Penh, an indicator of fibrosis) in both DEC therapy groups: 1 × 10^6^ dose (99.60% ± 7.22) and 5 × 10^6^ DEC dose (93.39% ± 3.00), when compared to vehicle-injected controls (107.26% ± 1.65) for which an increase of Penh was observed confirming a natural disease progression in the untreated mice and decreased diaphragm fibrosis after DEC therapy (Fig. 7A).

In addition, at 180 days, the time of expiration (Te) was reduced in DEC-injected mice in both: 1 × 10^6^ DEC (109.59 s ± 2.51) dose and was significant in 5 × 10^6^ DEC dose (95.31 s ± 0.63) due to reduced diaphragm pathology leading to amelioration of passive exhalation after DEC therapy when compared to the vehicle-injected controls (113.33 s ± 3.96). A significantly lower Te value was observed in 5 × 10^6^ DEC dose compared to 1 × 10^6^ DEC dose confirming a dose-dependent protective effect of DEC therapy on pulmonary function (Fig. 7B).

### Administration of Human DEC Therapy Improves Skeletal Muscle Function at 180 days after Systemic DEC Administration

Deterioration of the skeletal muscle function affects significantly daily activities and overall quality of life of DMD patients; thus, evaluation of muscle function is essential for assessment of disease progression. We have previously reported amelioration of gastrocnemius muscle function at 90 days after DEC therapy [41]. Here, we aimed to assess the long-term effect and dose response of human DEC on the grip strength and gastrocnemius muscle function assessed at 180-days after systemic-intraosseous administration of two doses of DEC cells (1 × 10^6^ and 5 × 10^6^) to the *mdx/scid* mice.

**Fig. 8:**
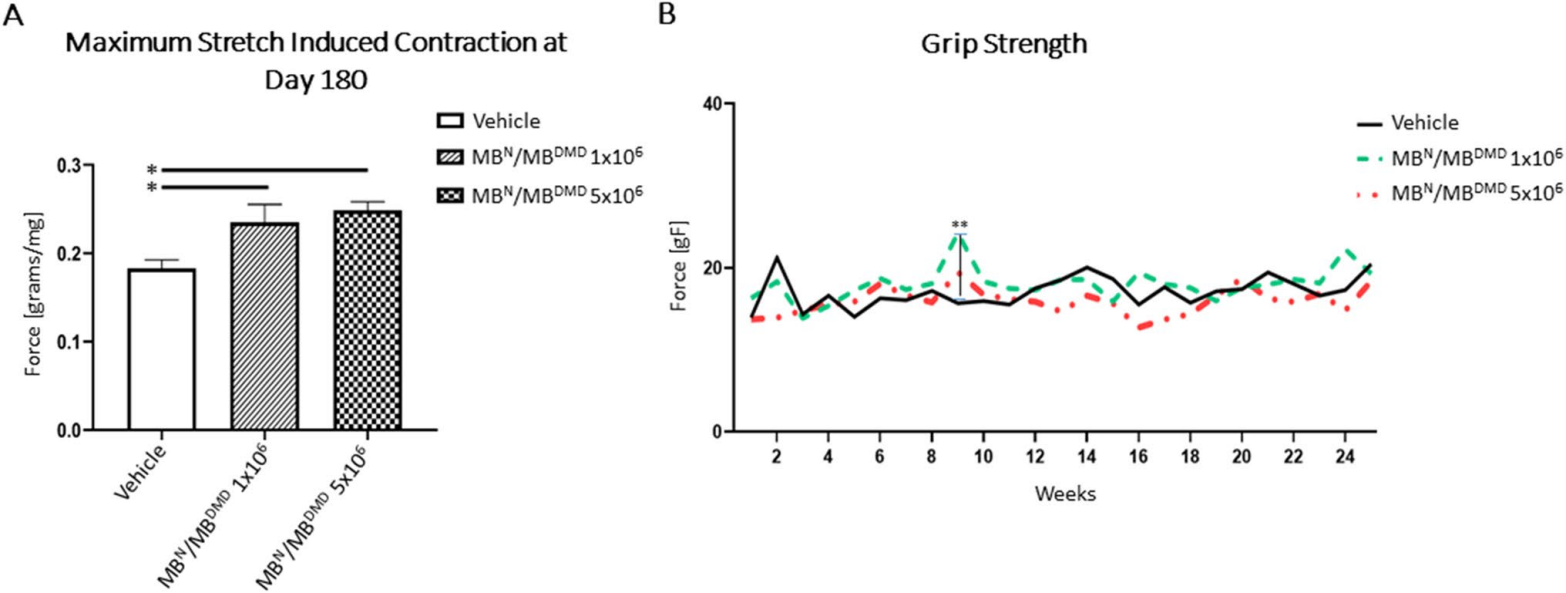
Human DEC therapy (1 × 10^6^ and 5 × 10^6^) improves skeletal muscle strength at 180 days after systemic-intraosseous administration to the *mdx/scid* mice. **(A)** At 180 days after DEC transplant the ex vivo Aurora muscle test identified a significant increase of maximum stretch-induced contraction of the gastrocnemius muscle in both DEC-injected groups compared to the vehicle-injected controls. Data presented as mean ± SEM; Aurora Scientific in vitro Muscle Test System. * *p* < 0.05. **(B)** Grip strength analysis at 180 days after systemic-intraosseous administration of DEC therapy revealed improved grip strength between 5–10 weeks follow-up period in both DEC-injected groups compared to the vehicle-injected controls, and significant grip strength improvement at 9-weeks in 1 × 10^6^ DEC-injected group when compared to the vehicle- injected control. **p < 0.01. Data presented as mean ± SEM

A significant amelioration of skeletal muscle function was observed for the critical parameters assessed by Aurora ex vivo muscle force test (Fig. 8A), which revealed a dose-dependent increase of maximum stretch induced contraction of the GM muscle after administration of both doses of DEC, when compared to the vehicle-injected controls. The maximum stretch induced contraction was significantly increased in the 1 × 10^6^ DEC dose (0.23 g ± 0.02) and in 5 × 10^6^ DEC dose (0.25 g ± 0.01), when compared to the vehicle-injected controls (0.18 g ± 0.01) confirming the long-term, dose dependent improvement of gastrocnemius muscle strength after systemic administration of DEC therapy.

### DEC Therapy Improves Grip Strength at 180 days after Systemic-Intraosseous Administration

Deterioration of the skeletal muscle function in DMD patients represent the early signs of the disease, thus we assessed muscle strength in the *mdx/scid* mice by in vivo grip strength test which revealed improved performance after treatment with both doses of DEC cells when compared with the vehicle-injected controls and at weeks 5–10 and for 1 × 10^6^ DEC dose revealed: (week 5- 17.25 ± 1.36, week 6- 18.70 ± 2.57, week 7- 17.35 ± 2.62, week 8- 18.08 ± 7.00, week 9- 24.23 ± 3.44, week 10- 18.32 ± 3.86; and for 5 × 10^6^ DEC dose revealed: week 5- 15.86 ± 5.59, week 6- 18.06 ± 4.89, week 7- 16.65 ± 3.98, week 8- 15.79 ± 4.14, week 9- 19.46 ± 8.02, week 10- 16.72 ± 3.36 when compared to the vehicle-injected controls (week 5—14.02 ± 2.16, week 6—16.30 ± 3.56, week 7—16.05 ± 3.68, week 8—17.19 ± 6.29, week 9—15.66 ± 3.94, week 10—15.94 ± 6.10). Additionally, mice injected with 1 × 10^6^ DEC dose significantly improved grip strength of the right limb at 9-weeks (24.23 ± 3.44) when compared with the vehicle-injected controls (15.66% ± 3.94%) (Fig. 8B).

## Discussion

Duchenne Muscular Dystrophy is a severe, genetic disease, caused by the lack of functional dystrophin in the affected muscles. DMD is characterized by progressive course and premature death, usually due to cardiopulmonary complications [48, 49]. Despite promising outcomes of the preclinical studies and recent reports on new approaches targeting the genetic defect, there is still no cure for DMD [3, 50–52].

Current approaches to treat DMD focus on dystrophin restoration by replacing and/or repairing the mutated dystrophin genes or by application of cell-based therapies [1, 2]. Although some of the gene therapies based on the exon skipping such as eteplirsen and ataluren have been approved for clinical use and were successfully introduced to the market, they are only applicable to the limited number of patients with the specific gene mutations [53, 54]. Another drug, drisapersen was not approved by the FDA due to the poor treatment benefits and the uncertain safety profile [2, 55, 56]. The efficacy of AAV-based microdystrophin administration [19, 20] in DMD is still understudied, although a recent clinical trial proved safety and functional improvement after intravenous delivery (www.clinicaltrials.gov/ct2/show/NCT03375164) [57, 58]. However, there are current reports on the immune response against the viral vectors, which is of a significant safety concern and limits repeated dosing of microdystrophin therapy [24, 58]. The CRISPR/Cas9 approach was aimed to restore dystrophin defect and revealed a broad range of dystrophin gene corrections in the experimental studies [14], however, this approach is not yet clinically applicable [13, 17, 50, 59]. Moreover, therapies involving genetic modification and viral vector application require a thorough preclinical testing, since low efficacy, tumorigenesis, sensitization and “off-target” mutations are still of a significant concern.

In the light of the limitations of gene therapies, the stem cell-based therapies are considered as the most promising therapeutic method for DMD patients. This approach aims for the restoration of dystrophin by distribution of normal, dystrophin expressing cells to the DMD affected muscles. Initial studies confirmed safety of human myoblast and stem cells transplantation, however, a major drawback was the immune response resulting in low efficacy of engraftment and the need for immunosuppressive therapy, to prevent myoblasts rejection [23, 26, 60, 61].

Only a few cell populations with myogenic properties and regenerative potential were investigated as a source for cell-based therapies in DMD and the satellite cells, muscle-derived stem cells and mesenchymal stem cells were found to be the most promising [62–66]. Initial studies testing satellite cells (SCs) confirmed dystrophin expression and improvement of muscle function in dystrophic mice, however, autologous transplantation of SCs was not clinically efficient due to insufficient engraftment and low myogenic potential [26]. Autologous transplantation of mesoangioblasts with PiggyBac transposon vector, enabled persistent full-length dystrophin expression by stable integration of DNA cargo into the cell genome in *mdx/scid* mouse model, however, the level of dystrophin expression was low, and amelioration of dystrophic muscle function has not been proven in the in vivo studies [60].

One of the most important factors determining efficacy of myoblast-based therapy is the route of cell administration and long-term engraftment. Previous studies testing systemic, therapeutic effect of mesoangioblasts, cardiomyocytes, MSC and iPS, investigated the intravenous, intramuscular, intracardiac and intraarterial routes of delivery with different efficacy results [23, 25, 34, 67–69].

Interestingly, the intraarterial and intravenous delivery of cardiomyocytes representing the striated muscles and cardiosphere-derived cells confirmed improvement of cardiac function as well as elicited the off-cardiac effects confirmed by improvement of the upper limb function, thus supporting the rationale for systemic delivery of cells of muscle tissue origin [69, 70].

We previously confirmed higher engraftment rates and better efficacy of chimeric cells following the systemic-intraosseous cells administration when compared with intravenous route of cell delivery [35, 71].

Moreover, we reported better engraftment of our Dystrophin Expressing Chimeric (DEC) therapy, after systemic intraosseous administration, when compared to the local intramuscular DEC delivery [39–41] and intravenous delivery (unpublished data). Similar findings of higher engraftment rate and amelioration of multi-organ function were confirmed by other investigators following intraosseous administration of bone marrow, cord-blood cells and MSC tested in clinical trials and treatment of blood disorders in both, the adult and pediatric patients’ population [72–76]. Recent Phase I clinical trial confirmed better efficacy of engraftment and safety after intra-bone co-administration of cord blood cells and MSC [77].

Until now, problems of limited engraftment, low dystrophin expression, limited cell survival and the need for immunosuppression remain unsolved and limit the routine clinical application of cell-based therapies. Thus, we have developed more effective approaches including systemic-intraosseous route of cell delivery for enhancement and maintenance of cell engraftment [37].

Furthermore, to improve engraftment without the need for immunosuppressive therapy, we have created chimeric cells of hematopoietic, mesenchymal and myoblast stem cell lineages of murine and human origin [35, 38, 39, 41]. We developed human Dystrophin Expressing Chimeric (DEC) cell lines via ex vivo fusion of human myoblasts (MB) derived from normal and DMD affected donors (MB^N^/MB^DMD^). Intramuscular administration of human DEC tested in preclinical studies in the mdx and *mdx/scid* mice models confirmed high myogenic potential which correlated with increased dystrophin expression and improved function [40]. Thus, to make this therapeutic approach more clinically relevant, we assessed the effect of systemic-intraosseous administration of human DEC and demonstrated long-term engraftment which correlated with increased dystrophin expression, reduced muscle pathology and improved function of cardiac, pulmonary and skeletal muscles at 90 days after systemic DEC transplant [41].

In the current study, considering the dynamic progression of DMD, our goal was to assess the long-term systemic effect of human DEC therapy for potential clinical applications. Moreover, considering the fact that clinical reports on assessment of current DMD therapies are tested in children of age 4–7-12 years old [78, 79] before development of severe cardiac or pulmonary problems, thus our goal in current study was to assess the protective effect of DEC therapy before severe progression of the disease and before development of the overt DMD -related cardiomyopathy or pulmonary failure, since it would be difficult to expect that either steroids, gene or cell-based therapies will reverse the organs failure and progression of the DMD disease Thus, the current preclinical study was performed on 6–8-weeks old *mdx/scid* mice which were observed up to 26 weeks after intraosseous DEC transplant. When considering the correlation of the age of tested animals and the length of the preclinical study with the future clinical trials in humans, it is estimated that based on this study the conversion of the age of mice to humans corresponds to the human age span from children to adults (7–20 years of age) and covers the age of DMD patients which will be considered in the future clinical trials [80, 81]. Thus, the reported here findings are encouraging since they may be relevant to the outcomes of future clinical studies in the DMD patients.

Since restoration of dystrophin is the major goal of all current therapies tested for DMD, in this report we have confirmed increased dystrophin expression in the heart, diaphragm and gastrocnemius muscle at 180 days after systemic administration of DEC to the *mdx/scid* mice. The increase in dystrophin expression was accompanied by amelioration of muscle pathology revealed by reduced fibrosis and inflammation in the assessed target organs of the DEC-injected mice. Specifically, at the histological level assessment of heart samples confirmed significantly reduced fibrosis, improved midventricular myocardium morphology, less prominent necrosis and decreased number of inflammatory cell infiltrates. Furthermore, amelioration of inflammation and fibrosis as well as normalization of muscle fiber size was observed in the diaphragm, and gastrocnemius muscle further confirming the multiorgan effect of DEC therapy. Moreover, DEC cell migration and long-term engraftment to the heart, diaphragm and gastrocnemius muscle of the *mdx* host was confirmed by expression of spectrin—the human-specific membrane protein marker**,** further confirming human origin of the engrafted cells at 180 days after systemic intraosseous DEC administration.

These findings are of clinical importance, since reports on histopathological assessments reveled that the fibro-fatty replacement of cardiomyocytes is a significant pathophysiological mechanism in the development of cardiomyopathy in the *mdx* mice [7]. Currently only few DMD clinical studies including assessment of givinostat therapy, reported a positive effect on muscle fiber regeneration, reduced fibrosis, and decreased muscle necrosis, however that study has not confirmed functional efficacy of the therapy [7].

In the current study, we confirmed a complimentary, multi-level effect of DEC therapy which was observed at both, the tissue level and the functional level, as confirmed by improvement of cardiac, respiratory and skeletal muscle pathology and function maintained up to 180 days after systemic DEC administration. It should be emphasized that the reported here long-term protective effect was maintained after a single dose of DEC therapy. These observations are clinically relevant, since studies assessing cardiomyopathy linked to DMD in humans, revealed that functional decline in progressive cardiopulmonary is characterized by the thinned wall thickness of the left ventricular (LV) and a progressive decrease in the ejection fraction and fractional shortening [81]. Thus, to assess the long-term efficacy of DEC therapy on cardiac function, we monitored echocardiographic changes over the entire study follow-up and confirmed both, the maintenance of the LV function at the baseline level up to 180 days post-transplant, as well as the dose dependent long-term improvement of the ejection fraction and fractional shortening recorded at 180-days after DEC administration.

In contrast, the vehicle-injected controls showed gradual decline of the left ventricular (LV) function leading to the significant drop in the ejection fraction and fractional shortening at 180-days after DEC transplant. Furthermore, there was a dose-dependent increase of the values of LV functional parameters of the stroke volume and cardiac output between the baseline and 180-day endpoint observed after administration of the higher DEC dose of 5 × 10^6^ cells, further confirming long-term protective effect of DEC therapy, since these parameters normally decline with age in the *mdx* mouse model [80]. In addition, the functional improvements recorded by echocardiography correlated with the reduced cardiac muscle pathology evidenced by reduced fibrosis and inflammation which are considered the leading mechanism of development of cardiomyopathy [82–84].

Interestingly, the dose-dependent increase of ejection fraction was observed also at 90 days after DEC administration, which highlights differences between the current study and our previous report [41], where the lower DEC doses (0.5 × 10^6^ and 1 × 10^6^) did not show dose dependent effect on cardiac function.

Importantly, the long-term improvement in cardiac function correlated with the improvement of respiratory function represented by the reduction of the enhanced pause (Penh) and time of expiration (Te) confirmed by plethysmography at 180-days after DEC transplant.

Finally, the analysis of long-term effect of human DEC therapy on the functional outcomes of skeletal muscle revealed increase in the maximum stretch induced contraction of the gastrocnemius muscle at 180-day study endpoint further confirming long-term multi-organ protective effect of DEC therapy after systemic-intraosseous administration.

There are several limitations of the study which should be addressed. We have based our study on the literature review of cardiac assessments by echocardiography [46, 85, 86] and MRI [87] and plethysmography [88, 89] reported by other investigators, where the number of evaluated animals (4–5) was similar to our study. Moreover, echocardiography and plethysmography assessments in mdx mice models were established in the independent laboratories by experienced investigators [39, 41] and were comparable with echocardiography [90] and plethysmography reports of other investigators [82, 88, 89, 91].

It should be noted that we have tested DEC therapy in the immunocompromised animal model since it allows for assessment of engraftment and efficacy of cells of human origin, but limits evaluation of the potential immune response. However, we have addressed this limitation in our previous work and have proven in the immunocompetent *mdx* mouse model that increased dystrophin expression correlated with significant improvement of skeletal and cardiac muscle function after intramuscular as well as intraosseous DEC administration, without evidence of side effects and without the need for immunosuppression [38–41]. Furthermore, the *mdx/scid* mouse model of DMD provides opportunity to test human DEC therapy created from normal and DMD affected donors according to the same manufacturing protocol which will be tested in the clinical trials. This fulfills regulatory requirements for the ex-vivo engineered DEC therapy as the novel, Advanced Therapy Medicinal Product (ATMP) [41]. Other investigators used immunocompromised NOD-*scid* mice model in the preclinical studies validating new ATMP of human skin-derived ABCB5 -positive mesenchymal stromal cells for clinical application (https://clinicaltrials.gov/ct2/show/NCT04971161) [67].

It is important to highlight, that functional improvements assessed in heart, diaphragm, and gastrocnemius muscle by standard functional tests of echocardiography, plethysmography and muscle strength tests, correlated with reduced muscle pathology parameters showing evidence of reduced interstitial fibrosis and inflammation, a trend towards normalization of the muscle fiber size, and decreased percentage of the centrally nucleated fibers in diaphragm and GM suggesting normalization of *mdx* muscle pathology in the selected DMD-affected organs at 180 days after systemic DEC transplant.

It should be emphasized, that despite the natural progression of DMD disease in the aging *mdx* mouse, the proven efficacy and the protective effect of DEC therapy over the long-term follow-up period of this study ranging between 6 and 26 weeks after DEC administration, represents valuable and clinically relevant findings, confirming both, the maintenance of the selected morphological and functional parameters at the baseline levels over the entire study follow-up, indicating the hold on the progression of the DMD disease, as well as the improvement of the important parameters of cardiac, respiratory and skeletal muscle function, confirming the long-term therapeutic effect of DEC therapy on the *mdx* pathology.

## Conclusions

In this study we have tested the long-term efficacy of DEC therapy by assessment of morphological, pathological and functional changes in the selected DMD-affected organs of heart, diaphragm and skeletal muscles. We confirmed that systemic-intraosseous administration of human DEC therapy restored dystrophin expression, which correlated with improved muscle morphology and pathology and long-term amelioration of function of the heart, respiratory and skeletal muscles as confirmed by standard echocardiography, plethysmography and muscle strength tests. DEC therapy does not require immunosuppression, is not limited to the specific gene mutation, does not cause sensitization and as such is universal for all DMD patients. These attributes make DEC therapy unique among other therapeutic approaches for DMD. Moreover, the long-term (180 days) protective effect of DEC on functional and morphological outcomes assessed in the most severely affected by DMD organs of heart, diaphragm and limb skeletal muscles, supports future clinical application of DEC as a safer and potentially more efficacious therapeutic approach applicable to all DMD patients regardless of gene mutation.

To the best of our knowledge, this is the first report assessing the long-term, multi-organ improvement after systemic administration of human DEC therapy.

This encouraging preclinical data introduces human DEC as a novel and universal therapeutic modality of the Advanced Therapy Medicinal Product (ATMP) with the potential to improve or halt progression of the disease and enhance quality of life of DMD patients.

## Supplementary Information

Below is the link to the electronic supplementary material.

Suppl Fig. 1 (3.57 MB)

High resolution image (TIF 836 KB)

## Data Availability

All data generated in this study are presented in the manuscript and are available for presentation upon request.

## Abbreviations

AAV: Adeno-Associated Virus
CNF: Centrally Nucleated Fiber
CO: Cardiac Output
DEC Cells: Dystrophin Expressing Chimeric Cells
DMD: Duchenne Muscular Dystrophy
EF: Ejection Fraction
FS: Fractional Shortening
GM: Gastrocnemius Muscle
H&E: Hematoxylin & Eosin
IF: Immunofluorescence
LV: Left Ventricular
LVEDD: Left Ventricular End Diastolic Diameter
LVEDV: Left Ventricular End Diastolic Volume
LVESD: Left Ventricular End Systolic Diameter
LVESV: Left Ventricular End Systolic Volume
MB: Myoblast
MB^DMD^: Duchenne Muscular Dystrophy Myoblast
MB^N^: Normal Myoblast
*mdx/scid*: Immunodeficient *mdx* mouse
SCs: Satellite Cells
SEM: Standard Error of Mean
SV: Stroke Volume
Te: Time of Expiration
VCA: Vascularized Composite Allotransplantation
WT: Wild Type

## References

[1] Sun C, Serra C, Lee G, Wagner KR (2019). Stem cell-based therapies for Duchenne muscular dystrophy. Experimental Neurology.

[2] Verhaart IEC, Aartsma-Rus A (2019). Therapeutic developments for Duchenne muscular dystrophy. Nature Reviews Neurology.

[3] Muir LA, Murry CE, Chamberlain JS (2016). Prosurvival factors improve functional engraftment of myogenically converted dermal cells into dystrophic skeletal muscle. Stem Cells and Development.

[4] Strehle EM, Straub V (2015). Recent advances in the management of Duchenne muscular dystrophy. Archives of Disease in Childhood.

[5] Buyse GM, Goemans N, Van Den Hauwe M, Meier T (2013). Effects of glucocorticoids and idebenone on respiratory function in patients with duchenne muscular dystrophy. Pediatric Pulmonology.

[6] Finder JD, Birnkrant D, Carl J, Farber HJ, Gozal D, Iannaccone ST (2004). Respiratory care of the patient with duchenne muscular dystrophy: ATS consensus statement. American Journal of Respiratory and Critical Care Medicine.

[7] Bettica P, Petrini S, D’Oria V, D’Amico A, Catteruccia M, Pane M (2016). Histological effects of givinostat in boys with Duchenne muscular dystrophy. Neuromuscular Disorders.

[8] Goemans N, Buyse G (2014). Current treatment and management of dystrophinopathies. Current Treatment Options in Neurology.

[9] Govoni A, Magri F, Brajkovic S, Zanetta C, Faravelli I, Corti S (2013). Ongoing therapeutic trials and outcome measures for Duchenne muscular dystrophy. Cellular and Molecular Life Sciences.

[10] Rafael-Fortney JA, Chimanji NS, Schill KE, Martin CD, Murray JD, Ganguly R (2011). Early treatment with lisinopril and spironolactone preserves cardiac and skeletal muscle in duchenne muscular dystrophy mice. Circulation.

[11] Sienkiewicz D, Okurowska Zawada B, Paszko Patej G, Kawnik K, Kulak W (2015). Duchenne muscular dystrophy: Current cell therapies. Therapeutic Advances in Neurological Disorders.

[12] Malik V, Rodino-Klapac LR, Mendell JR (2012). Emerging drugs for Duchenne muscular dystrophy. Expert Opinion on Emerging Drugs.

[13] Doetschman T, Georgieva T (2017). Gene Editing with CRISPR/Cas9 RNA-Directed Nuclease. Circulation Research.

[14] Long C, McAnally JR, Shelton JM, Mireault AA, Bassel-Duby R, Olson EN (2014). Prevention of muscular dystrophy in mice by CRISPR/Cas9-mediated editing of germline DNA. Science (80-).

[15] Min YL, Bassel-Duby R, Olson EN (2019). CRISPR correction of duchenne muscular dystrophy. Annual Review of Medicine.

[16] Nelson CE, Hakim CH, Ousterout DG, Thakore PI, Moreb EA, Rivera RMC (2016). In vivo editing improves muscle function in mouse of DMD. Science (80-)..

[17] Gee, P., Xu, H., Hotta, A. (2017). Cellular reprogramming, genome editing, and alternative CRISPR Cas9 technologies for precise gene therapy of Duchenne Muscular Dystrophy. *Stem Cells International,**2017*(DMD). 10.1155/2017/8765154 PMC5451761 28607562

[18] Yu, L., Zhang, X., Yang, Y., Li, D., Tang, K., Zhao, Z., et al. (2020). Small-molecule activation of lysosomal TRP channels ameliorates Duchenne muscular dystrophy in mouse models. *Science Advances, 6*(6). 10.1126/sciadv.aaz2736 PMC7032923 32128386

[19] Duan D, Systemic AAV (2018). Micro-dystrophin gene therapy for Duchenne Muscular Dystrophy. Molecular Therapy.

[20] Le Guiner, C., Servais, L., Montus, M., Larcher, T., Fraysse, B., Moullec, S., et al. (2017). Long-term microdystrophin gene therapy is effective in a canine model of Duchenne muscular dystrophy. *Nature Communications, 8*(May). 10.1038/ncomms16105 PMC5537486 28742067

[21] Colella P, Ronzitti G, Mingozzi F (2018). Emerging issues in AAV-mediated In Vivo gene therapy. Molecular Theraphy. Methods and Clinical Development.

[22] Palmieri B, Tremblay JP, Daniele L (2010). Past, present and future of myoblast transplantation in the treatment of Duchenne muscular dystrophy. Pediatric Transplantation.

[23] Cossu G, Previtali SC, Napolitano S, Cicalese MP, Tedesco FS, Nicastro F (2015). Intra-arterial transplantation of HLA -matched donor mesoangioblasts in Duchenne muscular dystrophy. EMBO Molecular Medicine.

[24] Barthélémy F, Wein N (2018). Personalized gene and cell therapy for Duchenne Muscular Dystrophy. Neuromuscular Disorders.

[25] Biressi S, Filareto A, Rando TA (2020). Stem cell therapy for muscular dystrophies. The Journal of Clinical Investigation.

[26] Meregalli M, Farini A, Belicchi M, Parolini D, Cassinelli L, Razini P (2013). Perspectives of stem cell therapy in Duchenne muscular dystrophy. FEBS Journal.

[27] Sitzia C, Farini A, Jardim L, Razini P, Belicchi M, Cassinelli L (2016). Adaptive immune response impairs the efficacy of autologous transplantation of engineered stem cells in dystrophic dogs. Molecular Therapy.

[28] Goudenege S, Lebel C, Huot NB, Dufour C, Fujii I, Gekas J (2012). Myoblasts derived from normal hESCs and dystrophic hipscs efficiently fuse with existing muscle fibers following transplantation. Molecular Therapy.

[29] Meng J, Counsell JR, Reza M, Laval SH, Danos O, Thrasher A (2015). Autologous skeletal muscle derived cells expressing a novel functional dystrophin provide a potential therapy for Duchenne Muscular Dystrophy. Science and Reports.

[30] Nitahara-Kasahara Y, Hayashita-Kinoh H, Ohshima-Hosoyama S, Okada H, Wada-Maeda M, Nakamura A (2012). Long-term engraftment of multipotent mesenchymal stromal cells that differentiate to form myogenic cells in dogs with duchenne muscular dystrophy. Molecular Therapy.

[31] Noviello M, Tedesco FS, Bondanza A, Tonlorenzi R, Rosaria Carbone M, Gerli MFM (2014). Inflammation converts human mesoangioblasts into targets of alloreactive immune responses: Implications for allogeneic cell therapy of DMD. Molecular Therapy.

[32] Torrente Y, Belicchi M, Marchesi C, D’Antona G, Cogiamanian F, Pisati F (2007). Autologous transplantation of muscle-derived CD133+ stem cells in Duchenne muscle patients. Cell Transplantation.

[33] Zhang Y, Zhu Y, Li Y, Cao J, Zhang H, Chen M (2015). Long-term engraftment of myogenic progenitors from adipose-derived stem cells and muscle regeneration in dystrophic mice. Human Molecular Genetics.

[34] Skuk D, Tremblay JP (2014). First study of intra-arterial delivery of myogenic mononuclear cells to skeletal muscles in primates. Cell Transplant..

[35] Hivelin M, Klimczak A, Cwykiel J, Sonmez E, Nasir S, Gatherwright J (2016). Immunomodulatory effects of different cellular therapies of bone marrow origin on chimerism induction and maintenance across MHC barriers in a face allotransplantation model. Archivum Immunolgiae et Therapiae Experimentalis.

[36] Siemionow M, Demir Y, Mukherjee A, Klimczak A (2005). Development and maintenance of donor-specific chimerism in semi-allogenic and fully major histocompatibility complex mismatched facial allograft transplants. Transplantation.

[37] Siemionow MZ, Klimczak A, Unal S (2005). Different routes of donor-derived hematopoietic stem cell transplantation for donor-specific chimerism induction across MHC barrier. Transplantation Proceedings.

[38] Siemionow M, Cwykiel J, Heydemann A, Garcia J, Marchese E, Siemionow K (2018). Creation of dystrophin expressing chimeric cells of myoblast origin as a novel stem cell based therapy for Duchenne Muscular Dystrophy. Stem Cell Rev Reports..

[39] Siemionow M, Malik M, Langa P, Cwykiel J, Brodowska S, Heydemann A (2019). Cardiac protection after systemic transplant of Dystrophin Expressing Chimeric (DEC) cells to the mdx mouse model of Duchenne Muscular Dystrophy. Stem Cell Rev Reports..

[40] Siemionow M, Cwykiel J, Heydemann A, Garcia J, Marchese E, Siemionow K (2018). Dystrophin Expressing Chimeric (DEC) human cells provide a potential therapy for Duchenne Muscular Dystrophy. Stem Cell Rev Reports..

[41] Siemionow M, Langa P, Harasymczuk M, Cwykiel J, Sielewicz M, Smieszek J, et al. Human dystrophin expressing chimeric (DEC) cell therapy ameliorates cardiac, respiratory, and skeletal muscle’s function in Duchenne muscular dystrophy. Stem Cells Translational Medicine 2021;(July):1406–18. 10.1002/sctm.21-0054 PMC8459641 34291884

[42] Siemionow M, Rampazzo A, Bassiri B, Cwykiel J, Klimczak A, Madajka M (2016). The reversed paradigm of chimerism induction: Donor conditioning with recipient-derived bone marrow cells as a novel approach for tolerance induction in vascularized composite allotransplantation. Microsurgery.

[43] Siemionow M, Papay F, Alam D, Bernard S, Djohan R, Gordon C (2009). Near-total human face transplantation for a severely disfigured patient in the USA. Lancet.

[44] Siemionow, M. Z., Papay, F., Djohan, R., Bernard, S., Gordon, C. R., Alam, D., et al. (2010). First U.S. near-total human face transplantation: A paradigm shift for massive complex injuries. *Plastic and Reconstructive Surgery, 125*(1):111–22. 10.1097/PRS.0b013e3181c15c4c 19770815

[45] Fayssoil A, Renault G, Guerchet N, Marchiol-Fournigault C, Fougerousse F, Richard I (2013). Cardiac characterization of mdx mice using high-resolution doppler echocardiography. Journal of Ultrasound in Medicine.

[46] Bondoc AB, Detombe S, Dunmore-Buyze J, Gutpell KM, Liu L, Kaszuba A (2014). Application of 3-D echocardiography and gated micro-computed tomography to assess cardiomyopathy in a mouse model of Duchenne Muscular Dystrophy. Ultrasound in Medicine and Biology.

[47] Roberts, N. W., Holley-Cuthrell, J., Gonzalez-Vega, M., Mull, A. J., Heydemann, A. (2015) Biochemical and functional comparisons of mdx and Sgcg -/- muscular dystrophy mouse models. *BioMed Research International, 2015*(2). 10.1155/2015/131436 PMC4433636 26064876

[48] Birnkrant DJ, Bushby K, Bann CM, Apkon SD, Blackwell A, Brumbaugh D (2018). Diagnosis and management of Duchenne muscular dystrophy, part 1: Diagnosis, and neuromuscular, rehabilitation, endocrine, and gastrointestinal and nutritional management. Lancet Neurology.

[49] Van Ruiten HJA, Marini Bettolo C, Cheetham T, Eagle M, Lochmuller H, Straub V (2016). Why are some patients with Duchenne muscular dystrophy dying young: An analysis of causes of death in North East England. European Journal of Paediatric Neurology.

[50] Li HL, Fujimoto N, Sasakawa N, Shirai S, Ohkame T, Sakuma T (2015). Precise correction of the dystrophin gene in duchenne muscular dystrophy patient induced pluripotent stem cells by TALEN and CRISPR-Cas9. Stem Cell Reports.

[51] Łoboda, A., Dulak, J. (2020). *Muscle and cardiac therapeutic strategies for Duchenne muscular dystrophy: past, present, and future* (Vol. 72, pp. 1227–1263), Pharmacological Reports. Springer International Publishing. 10.1007/s43440-020-00134-x 10.1007/s43440-020-00134-x PMC7550322 32691346

[52] Pascual-Morena C, Cavero-Redondo I, Álvarez-Bueno C, Mesas AE, Pozuelo-Carrascosa D, Martínez-Vizcaíno V (2020). Restorative treatments of dystrophin expression in Duchenne muscular dystrophy: A systematic review. Annals of Clinical Translational Neurology.

[53] Randeree L, Eslick GD (2018). Eteplirsen for paediatric patients with Duchenne muscular dystrophy: A pooled-analysis. Journal of Clinical Neuroscience.

[54] Campbell C, Barohn RJ, Bertini E, Chabrol B, Pietro CG, Darras BT (2020). Meta-analyses of ataluren randomized controlled trials in nonsense mutation Duchenne muscular dystrophy. Journal of Comparative Effectiveness Research.

[55] Goemans NM, Tulinius M, Van Den Hauwe M, Kroksmark AK, Buyse G, Wilson RJ (2016). Long-term efficacy, safety, and pharmacokinetics of drisapersen in duchenne muscular dystrophy: Results from an open-label extension study. PLoS ONE.

[56] Goemans N, Mercuri E, Belousova E, Komaki H, Dubrovsky A, McDonald CM (2018). A randomized placebo-controlled phase 3 trial of an antisense oligonucleotide, drisapersen, in duchenne muscular dystrophy. Neuromuscular Disorders.

[57] Mendell JR, Al-Zaidy SA, Rodino-Klapac LR, Goodspeed K, Gray SJ, Kay CN (2021). Current clinical applications of in vivo gene therapy with AAVs. Molecular Therapy.

[58] Mendell JR, Sahenk Z, Lehman K, Nease C, Lowes LP, Miller NF (2020). Assessment of systemic delivery of rAAVrh74.MHCK7.micro-dystrophin in children with Duchenne Muscular Dystrophy: A nonrandomized controlled trial. JAMA Neurology.

[59] Chemello F, Bassel-Duby R, Olson EN (2020). Correction of muscular dystrophies by CRISPR gene editing. The Journal of Clinical Investigation.

[60] Iyer PS, Mavoungou LO, Ronzoni F, Zemla J, Schmid-Siegert E, Antonini S (2018). autologous cell therapy approach for Duchenne Muscular Dystrophy using PiggyBac Transposons and Mesoangioblasts. Molecular Therapy.

[61] Sun, C., Choi, I. Y., Rovira Gonzalez, Y. I., Andersen, P., Conover Talbot, C., Iyer, S. R., et al. (2020). Duchenne muscular dystrophy hiPSC–derived myoblast drug screen identifies compounds that ameliorate disease in mdx mice. *JCI Insight, 5*(11). 10.1172/jci.insight.134287 PMC7308059 32343677

[62] Bier A, Berenstein P, Kronfeld N, Morgoulis D, Ziv-Av A, Goldstein H (2018). Placenta-derived mesenchymal stromal cells and their exosomes exert therapeutic effects in Duchenne muscular dystrophy. Biomaterials.

[63] Danisovic L, Culenova M, Csobonyeiova M (2018). Induced pluripotent stem cells for Duchenne Muscular Dystrophy modeling and therapy. Cells.

[64] He R, Li H, Wang L, Li Y, Zhang Y, Chen M (2020). Engraftment of human induced pluripotent stem cell-derived myogenic progenitors restores dystrophin in mice with duchenne muscular dystrophy. Biological Research.

[65] Sampaolesi M, Blot S, D’Antona G, Granger N, Tonlorenzi R, Innocenzi A (2006). Mesoangioblast stem cells ameliorate muscle function in dystrophic dogs. Nature.

[66] Tappenbeck N, Schröder HM, Niebergall-Roth E, Hassinger F, Dehio U, Dieter K (2019). In vivo safety profile and biodistribution of GMP-manufactured human skin-derived ABCB5-positive mesenchymal stromal cells for use in clinical trials. Cytotherapy.

[67] Kerstan A, Niebergall-Roth E, Esterlechner J, Schröder HM, Gasser M (2021). Ex vivo-expanded highly pure ABCB5 + mesenchymal stromal cells as Good Manufacturing Practice-compliant autologous advanced therapy medicinal product for clinical use: process validation and first in-human data. Cytotherapy.

[68] Nakamura A (2017). Moving towards successful exon-skipping therapy for Duchenne muscular dystrophy. Journal of Human Genetics.

[69] Taylor M, Jefferies J, Byrne B, Lima J, Ambale-Venkatesh B, Ostovaneh MR (2019). Cardiac and skeletal muscle effects in the randomized HOPE-Duchenne trial. Neurology.

[70] McDonald CM, Marbán E, Hendrix S, Hogan N, Ruckdeschel Smith R, Eagle M, Finkel RS, Tian C, Janas J, Harmelink MM, Varadhachary AS, Taylor MD, Hor KN, Mayer OH, Henricson EK, Furlong P, Ascheim DD, Rogy S, Williams P, Marbán L, HOPE-2 Study Group (2022). Repeated intravenous cardiosphere-derived cell therapy in late-stage Duchenne muscular dystrophy (HOPE-2): a multicentre, randomised, double-blind, placebo-controlled, phase 2 trial. Lancet..

[71] Siemionow M, Zielinski M, Ozmen S, Izycki D (2005). Intraosseus transplantation of donor-derived hematopoietic stem and progenitor cells induces donor-specific chimerism and extends composite tissue allograft survival. Transplantation Proceedings.

[72] Bonifazi F, Dan E, Labopin M, Sessa M, Guadagnuolo V, Ferioli M (2019). Intrabone transplant provides full stemness of cord blood stem cells with fast hematopoietic recovery and low GVHD rate: Results from a prospective study. Bone Marrow Transplantation.

[73] Frassoni F, Gualandi F, Podestà M, Raiola AM, Ibatici A, Piaggio G (2008). Direct intrabone transplant of unrelated cord-blood cells in acute leukaemia: A phase I/II study. The lancet Oncology.

[74] Goto T, Murata M, Terakura S, Nishida T, Adachi Y, Ushijima Y (2018). Phase i study of cord blood transplantation with intrabone marrow injection of mesenchymal stem cells. Medecine (United States)..

[75] Lee H, Park JB, Lee S, Baek S, Kim HS, Kim SJ (2013). Intra-osseous injection of donor mesenchymal stem cell (MSC) into the bone marrow in living donor kidney transplantation; a pilot study. Journal of Translational Medicine.

[76] Marktel S, Scaramuzza S, Cicalese MP, Giglio F, Galimberti S, Lidonnici MR (2019). Intrabone hematopoietic stem cell gene therapy for adult and pediatric patients affected by transfusion-dependent ß-thalassemia. Nature Medicine.

[77] Goto T, Murata M, Nishida T (2021). Phase I clinical trial of intra-bone marrow cotransplantation of mesenchymal stem cells in cord blood transplantation. Stem Cells Translational Medicine.

[78] Mah, J. K., Clemens, P. R., Guglieri, M., et al. (2022). Efficacy and safety of Vamorolone in Duchenne Muscular Dystrophy: A 30-month nonrandomized controlled open-label extension trial. *JAMA Network Open*, *5*(1):e2144178. Published 2022 Jan 4. doi:10.1001/jamanetworkopen.2021.44178 10.1001/jamanetworkopen.2021.44178 PMC8790668 35076703

[79] McDonald CM, Shieh PB, Abdel-Hamid HZ (2021). Open-label evaluation of eteplirsen in patients with Duchenne Muscular Dystrophy amenable to exon 51 skipping: PROMOVI trial. J Neuromuscul Dis..

[80] Dutta S, Sengupta P (2016). Men and mice: Relating their ages. Life Sciences.

[81] Wang, S., Lai, X., Deng, Y., Song, Y. (2020). Correlation between mouse age and human age in anti-tumor research: Significance and method establishment. *Life Sciences, 242*(December 2019). 10.1016/j.lfs.2019.117242 31891723

[82] Spurney CF, Knoblach S, Pistilli EE, Nagaraju K, Martin GR, Hoffman EP (2008). Dystrophin-deficient cardiomyopathy in mouse: Expression of Nox4 and Lox are associated with fibrosis and altered functional parameters in the heart. Neuromuscular Disorders.

[83] Verhaart IEC, van Duijn RJM, den Adel B, Roest AAW, Verschuuren JJGM, Aartsma-Rus A (2012). Assessment of cardiac function in three mouse dystrophinopathies by magnetic resonance imaging. Neuromuscular Disorders.

[84] Gallot YS, Straughn AR, Bohnert KR, Xiong G, Hindi SM, Kumar A (2018). MyD88 is required for satellite cell-mediated myofiber regeneration in dystrophin-deficient mdx mice. Human Molecular Genetics.

[85] Shin JH, Nitahara-Kasahara Y, Hayashita-Kinoh H, Ohshima-Hosoyama S, Kinoshita K, Chiyo T, Okada H, Okada T, Takeda S (2011). Improvement of cardiac fibrosis in dystrophic mice by rAAV9-mediated microdystrophin transduction. Gene Therapy.

[86] Andrews TG, Lindsey ML, Lange RA, Aune GJ (2014). Cardiac assessment in pediatric mice: Strain analysis as a diagnostic measurement. Echocardiography.

[87] Feintuch A, Zhu Y, Bishop J, Davidson L, Dazai J, Bruneau BG, Henkelman RM (2007). 4D cardiac MRI in the mouse. NMR in Biomedicine.

[88] Hawkins EC, Bettis AK, Kornegay JN (2020). Expiratory dysfunction in young dogs with golden retriever muscular dystrophy. Neuromuscular Disorders.

[89] Ishizaki M, Suga T, Kimura E, Shiota T, Kawano R, Uchida Y, Uchino K, Yamashita S, Maeda Y, Uchino M (2008). Mdx respiratory impairment following fibrosis of the diaphragm. Neuromuscular Disorders.

[90] Spurney CF, Sali A, Guerron AD, Iantorno M, Yu Q, Gordish-Dressman H, Rayavarapu S, van der Meulen J, Hoffman EP, Nagaraju K (2011). Losartan decreases cardiac muscle fibrosis and improves cardiac function in dystrophin-deficient mdx mice. Journal of Cardiovascular Pharmacology and Therapeutics.

[91] Yu Q, Morales M, Li N, Fritz AG, Ruobing R, Blaeser A, Francois E, Lu QL, Nagaraju K, Spurney CF (2018). Skeletal, cardiac, and respiratory muscle function and histopathology in the P448Lneo- mouse model of FKRP-deficient muscular dystrophy. Skelet Muscle..

