# Correction: Long-Term Protective Effect of Human Dystrophin Expressing Chimeric (DEC) Cell Therapy on Amelioration of Function of Cardiac, Respiratory and Skeletal Muscles in Duchenne Muscular Dystrophy

**DOI:** 10.1007/s12015-022-10472-3

**Published:** 2022-11-28

**Authors:** Maria Siemionow, Paulina Langa, Sonia Brodowska, Katarzyna Kozlowska, Kristina Zalants, Katarzyna Budzynska, Ahlke Heydemann

**Affiliations:** 1 Department of Orthopaedics, University of Illinois at Chicago, Chicago, IL USA; 2 Department of Surgery, Poznan University of Medical Science, Poznan, Poland; 3 Department of Physiology and Biophysics, University of Illinois at Chicago, Chicago, IL USA

**Correction: Stem Cell Reviews and Reports**

**https://doi.org/10.1007/s12015-022-10384-2**

The original online version of this article was revised: In this article, the wrong figure appeared as Fig. 5. The figure should have appeared as shown below
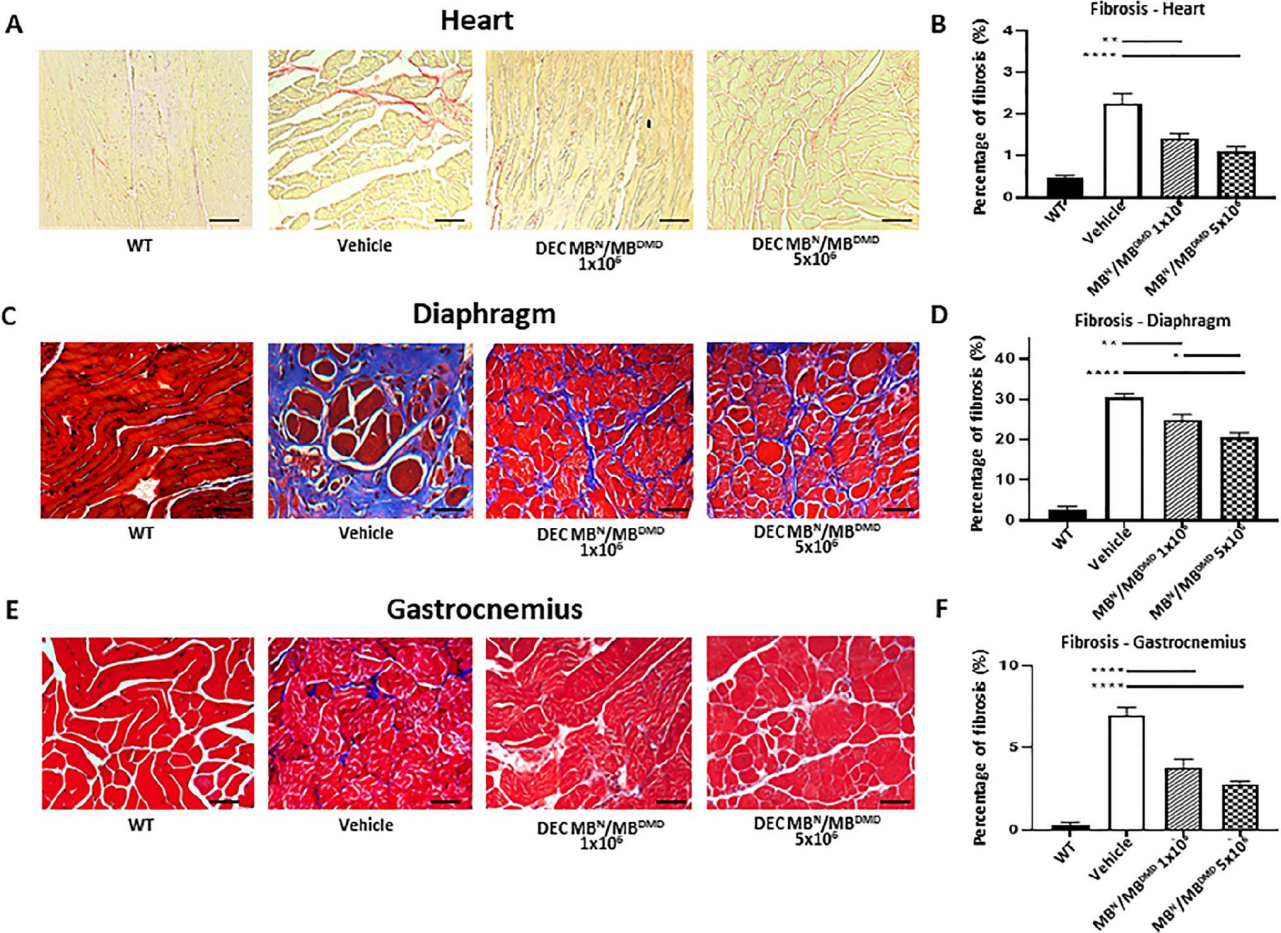


The original article has been corrected.

