# Supplementary figures and images for: Long-Term Protective Effect of Human Dystrophin Expressing Chimeric (DEC) Cell Therapy on Amelioration of Function of Cardiac, Respiratory and Skeletal Muscles in Duchenne Muscular Dystrophy
Source: Stem Cell Rev Rep. 2022 May 19;18(8):2872–92. doi: 10.1007/s12015-022-10384-2 (PMC9622520; doi:10.1007/s12015-022-10384-2)

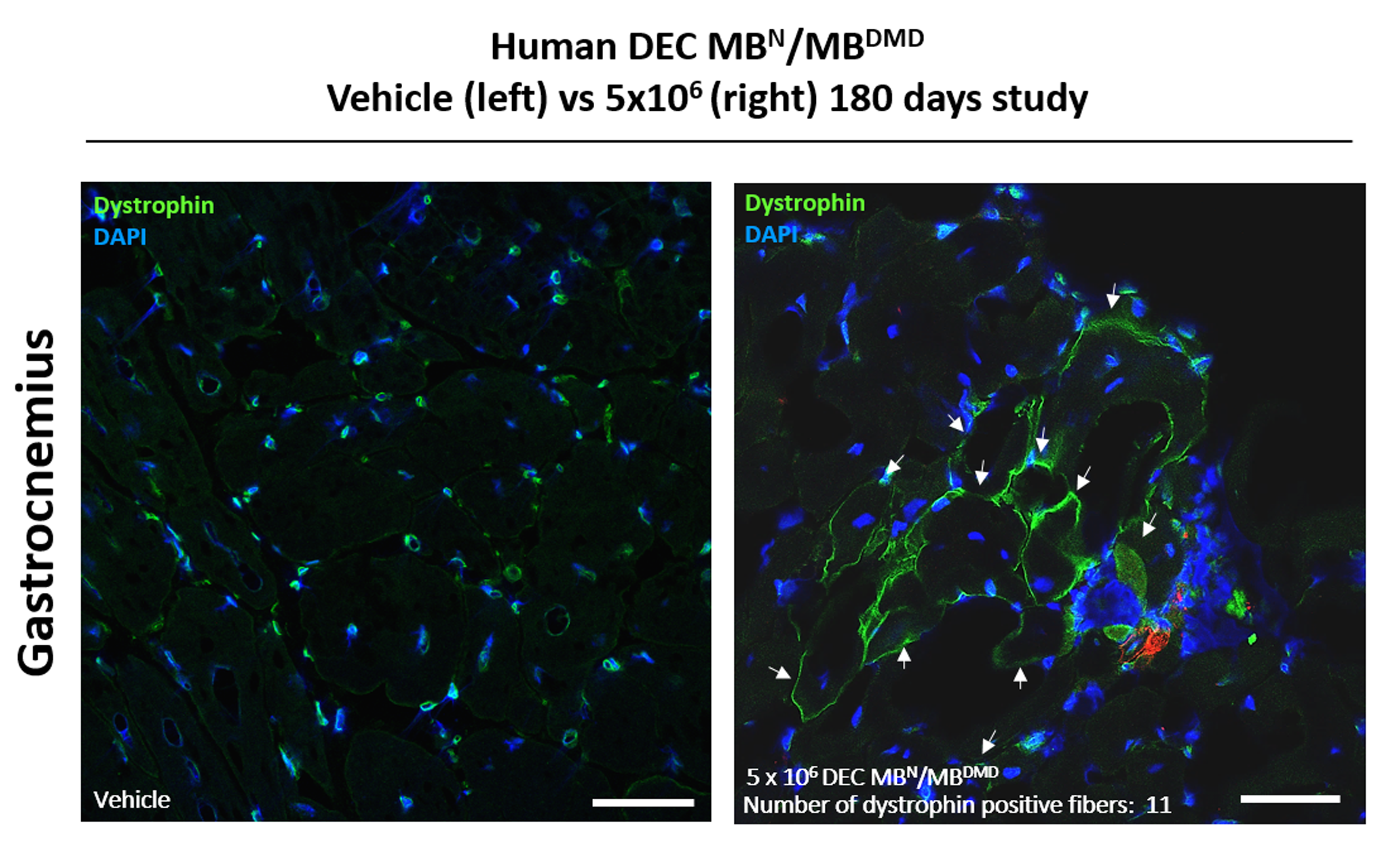

Supplement: Supplementary file 1 — Suppl Fig. 1 (3.57 MB) [file 12015_2022_10384_Fig9_ESM.png]

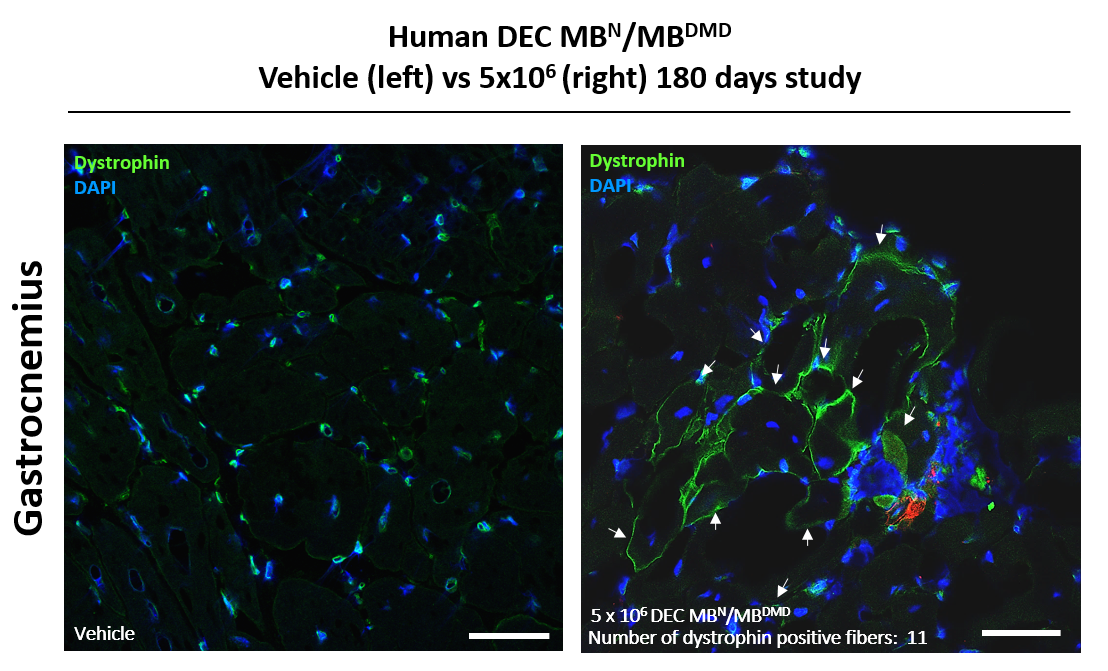

Supplement: Supplementary file 2 — High resolution image (TIF 836 KB) [file 12015_2022_10384_MOESM1_ESM.tif]
